# A Liquid-Core Fiber Platform for Classical and Entangled Two-Photon Absorption Measurements

**DOI:** 10.1021/acsphotonics.4c02076

**Published:** 2025-03-07

**Authors:** Kristen M. Parzuchowski, Michael D. Mazurek, Charles H. Camp, Martin J. Stevens, Ralph Jimenez

**Affiliations:** † JILA, University of Colorado Boulder, Boulder, Colorado 80309, United States; ‡ Department of Physics, University of Colorado Boulder, Boulder, Colorado 80309, United States; § Associate of the National Institute of Standards and Technology, Boulder, Colorado 80305, United States; ∥ National Institute of Standards and Technology, Gaithersburg, Maryland 20899, United States; ⊥ National Institute of Standards and Technology, Boulder, Colorado 80305, United States; # Department of Chemistry, University of Colorado Boulder, Boulder, Colorado 80309, United States

**Keywords:** two-photon absorption, spontaneous parametric downconversion, liquid-core fiber, cross-section, low power, fluorescence detection

## Abstract

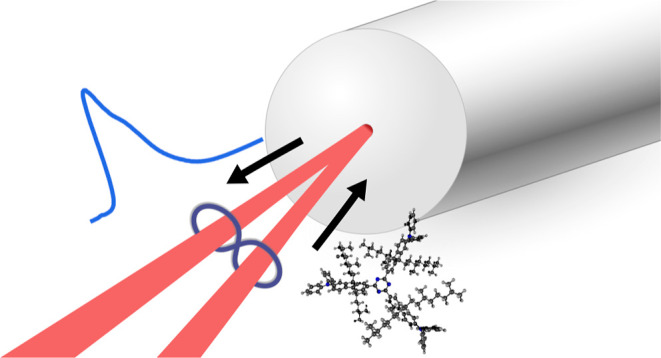

We introduce a toluene-filled fiber platform for two-photon absorption measurements. By confining both the light and molecular sample inside the 5 μm hollow core of the fiber, we increase the distance over which the nonlinear light–matter interaction occurs. With only a 7.3 nL excitation volume, we measure classical two-photon absorption (C2PA) at an average laser power as low as 1.75 nW, which is a 45-fold improvement over a conventional free-space technique. We use this platform to attempt to measure entangled two-photon absorption (E2PA), a process with a limited regime where the quantum advantage is large. This regime arises due to a crossover from linear to quadratic scaling with photon flux as photon flux is increased. Recently, several teams of researchers have reported that E2PA cross-sections are much smaller than previously claimed. As a result, the linear scaling dominates at photon fluxes so low that it is extremely difficult or impossible to measure using conventional free-space techniques. In this report, we implement the first E2PA measurement using a waveguide. We see no evidence of E2PA, and we set an upper bound on the cross-section consistent with these recent reports.

## Introduction

Liquid-core fibers (LCF) provide platforms for material characterization,^1^ optical limiting,^2^ sensing^3^ and light generation.^4,5^ These fibers are designed using a variety of geometries and materials that aid in their ability to confine light into micrometer-scale cores over centimeter-scale distances. The light reaches high intensities within LCF, which enables nonlinear processes to dominate^6,7^ and thus become easier to observe than in conventional free-space techniques. Processes like four-wave mixing, third-harmonic generation, supercontinuum generation, Raman scattering and two-photon absorption (2PA) have been targeted in LCF.

Two-photon absorption was first observed in capillary LCFs in the 1990s.^8,9^ These studies included models that were used to derive molecular 2PA coefficients from measurements of energy transmitted out of the fiber as a function of energy sent into the fiber.^10,11^ A number of 2PA fiber studies have followed these, including 2PA observed in liquid- or vapor-core photonic crystal fibers^12,13^ and using tapered or exposed-core fiber.^14,15^ One study in particular^12^ used a water-filled photonic-crystal-fiber system to measure a 2PA signal at analyte concentrations as low as 10^–9^ M (M = mol L^–1^), which is roughly 4 orders-of-magnitude smaller than typical concentrations used in free-space configurations. Although a one-to-one comparison of the two techniques was not performed, this report exemplifies how sensitive LCF techniques can lead to significant improvements in 2PA signal levels.

Here, we show that with a toluene-filled fiber, 2PA cross-sections, which are the quantities that describe the strength of the 2PA process, can be measured at extremely low powers. To the best of our knowledge, this work is the first to show a 2PA measurement at an average power as low as 1.75 nW. This is a 45-fold lower power than that achieved in our previous results^16^ for low-power 2PA in free space for the same sample. These fiber and free-space measurements serve as a one-to-one comparison since both were designed to test the sensitivity limits of the platforms. We believe this is the first time that the advantage of the fiber platform for 2PA has been quantified.

This sensitivity, paired with the ability to increase our laser power by up to 9 orders of magnitude, demonstrates that the platform is well equipped to boost previously undetectable signals. This is necessary, for example, for measurements of C2PA cross-sections at much less efficient wavelengths or for much less efficient absorbers. Furthermore, rather than being limited to a high peak power laser,^17^ unconventional light sources may produce sizable 2PA signals.

One such light source is an entangled photon pair source based on spontaneous parametric downconversion (SPDC). We employ an SPDC source in an attempt to measure entangled two-photon absorption (E2PA). To differentiate between E2PA and 2PA excited by a laser source, we refer to the latter process as classical two-photon absorption (C2PA). The E2PA process has been a subject of theoretical study since the 1980s (refs (18–20)), driven by the idea that the strong temporal and spatial correlations of SPDC photons are ideal for 2PA. These temporal and spatial correlations are characterized by the entanglement time and area, respectively.^20^ The entanglement time quantifies the width of the joint probability distribution of differences in arrival times of the two photons forming a pair, defined at the location of interest. The entanglement area is proportional to the width of the joint probability distribution of differences in positions of the two photons forming a pair, defined at the location of interest—to calculate entanglement area, the widths from the two transverse dimensions must be used.

The E2PA process is known to scale linearly with the excitation photon flux, rather than the quadratic scaling of C2PA, in the low-gain parametric downconversion (PDC) regime where, if it can be measured, it dominates over the quadratic component and thus demonstrates a quantum advantage. In the high-gain PDC regime, a quadratic scaling is recovered, with the possibility of up to 3-fold signal boost relative to excitation with a coherent laser source. This boost reflects a higher likelihood of two photons overlapping due to the increased statistical fluctuations of the excitation source. A more extensive description of the SPDC regimes and expected quantum advantage for various bandwidth conditions of the pump, SPDC and absorption spectrum is covered in refs (21 and 22).

Recently, a wide range of evidence from a variety of experimental research studies has shown that molecular E2PA cross-sections are much smaller than previously reported. The majority of studies have reported no E2PA signal above the noise floor in the low-gain (refs (22–32)) and moderate-gain PDC regimes (ref (16)). One study reported an extremely weak linear-scaling signal,^33^ however another study closely replicated this experiment and was unable to observe the reported signal.^22^ Two studies have reported quadratic-scaling signals using PDC (refs (22 and 31)). In the report by Raymer and co-workers,^22^ a high-gain PDC source is used and the resulting signal exhibits a moderate boost relative to a coherent source, which is consistent with the increased photon number fluctuations of the excitation source. In the report by Wang and co-workers,^31^ a low-gain PDC source is used to excite upconversion nanoparticles that have long-lived intermediate states and thus can be excited via uncorrelated photon pairs despite using an excitation source with significant temporal separation between photon pairs. These experimental reports are reinforced by recent theoretical works that show that the linear scaling will dominate at photon fluxes so low that the resulting minuscule signal will be difficult or impossible to measure with current technology (refs (21, 34–36)). Furthermore, the conditions under which the minuscule signal is maximized require careful selection of the SPDC source and two-photon absorber. These experimental and theoretical studies all consider conventional free-space excitation conditions.

Here we present, to the best of our knowledge, the first E2PA measurement implemented in a waveguide. Our approach uses a relatively high flux of photon pairs to maximize our likelihood of measuring a signal. In our setup, this high flux is generated by a moderate-gain PDC process. We do not observe E2PA and set an upper bound on the E2PA cross-section of the molecule AF455.

In this paper, we discuss the principles of our experimental scheme and the design considerations along with our experimental implementation and its characterization. Next, we present our results for excitation of the molecule AF455 with both laser and SPDC photons and derive the C2PA and E2PA cross-sections, respectively. Finally we compare our results with those from previous free-space measurements.^16^ More details on experimental characterization, data acquisition and data analysis are provided in the Supporting Information

## Operating Principles

We confine both a sample (two-photon-absorbing molecules in solution) and an excitation beam in the core of a hollow-core fiber. Here the goal is to excite the confined molecules by 2PA. The excited molecules sometimes emit fluorescence and some of that light is guided back out of the fiber and can be detected.

**Figure 1:**
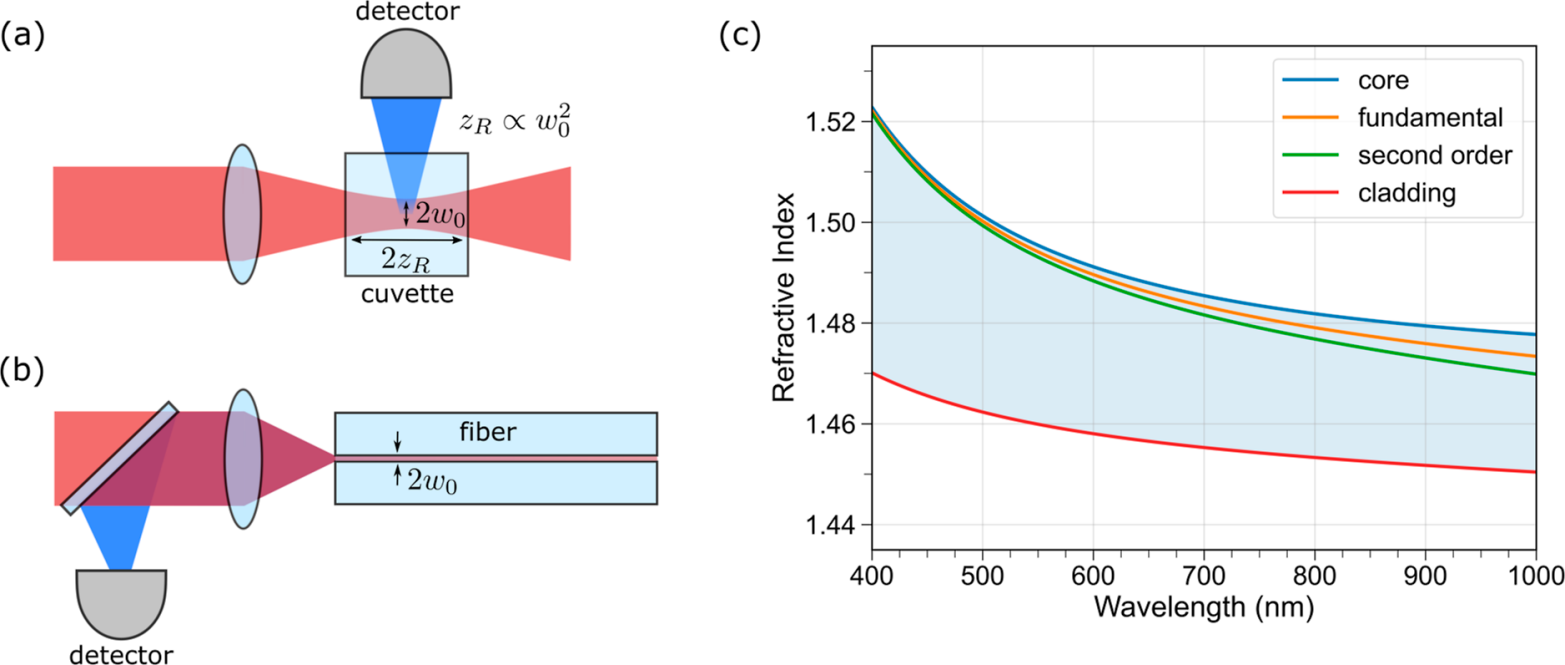
Illustration of (a) a conventional free-space and (b) a fiber-based 2PA measurement scheme. In the free-space approach, the light is focused into the cuvette to a beam waist *ω*_0_. The Rayleigh range *z*_R_ characterizes the distance over which the beam size is . When *ω*_0_ is decreased so is *z*_R_, thus high intensity beams can only be maintained over short distances. Fluorescence is generated primarily at the waist of the beam and the fraction emitted in the direction of the detector is collected. In the fiber-based approach, the light is focused into fiber with waist *ω*_0_ and maintained over long lengths. Fluorescence is generated along the length of the fiber and the fraction directed opposite of the excitation beam is collected on a detector. (c) Refractive index as a function of wavelength for the relevant media and first two modes of guided light. The toluene-filled core (blue) and silica cladding (red) indices are shown. The mode indices for the fundamental (orange) and second-order (green) modes are shown. Additional modes can propagate through the filled fiber with mode indices filling the blue shaded region between the second-order mode and cladding indices.

The benefit of this scheme is illustrated in Figure 1a,b. In the free-space approach (Figure 1a), light is focused into the cuvette to a waist *ω*_0_ and diverges on a length scale characterized by the Rayleigh range *z*_R_ which scales quadratically with *ω*_0_. Thus, increasing beam intensity occurs at the cost of maintaining the intensity over a shorter distance *z*_R_. For typical focusing conditions of *ω*_0_ ≈ 30 μm for an 810 nm beam into toluene, *z*_R_ ≈ 5.2 mm, which is about half of the cuvette’s length. In contrast, with the fiber-based approach (Figure 1b), the beam waist *ω*_0_ is maintained with low loss over the entire propagation length of the fiber because of optical confinement. Typically, *ω*_0_ is much smaller in fiber than in free space. This high light intensity is advantageous for 2PA because two photons must be spatially overlapped at a molecule for absorption to occur. Maintaining the peak intensity of the focused beam over centimeter length-scales increases the excitation probability because of the interaction with a large number of molecules.

To achieve broadband guidance of both the excitation and fluorescence photons, we selected our fiber for index guidance. In this regime, nearly all light traveling in the core and incident onto the core-cladding interface at an angle (with respect to the normal to the interface) equal to or greater than the critical angle, θ_c_ = arcsin (*n*_clad_/*n*_core_) where *n*_clad_ and *n*_core_ are the indices of refraction of the cladding and core of the fiber, is guided through the fiber by total internal reflection. It is likely that our cladding material is imperfect, due in part to optical losses and its finite extent, and results in a minor deviation from perfect total internal reflection. The critical angle is only a real number if the refractive index of the cladding is smaller than that of the core. To satisfy this criterion, we use a standard capillary tubing (inner diameter = 5 μm, outer diameter = 150 μm) with a silica cladding and fill the hollow core with toluene. The critical angle under these conditions is real for both the excitation and fluorescence wavelength regions. Many other common solvents, such as water, methanol or chloroform, have indices of refraction smaller than that of silica.

The refractive indices as a function of wavelength for the toluene core and silica cladding are plotted in Figure 1c using the known dispersion equations for the materials.^37,38^ The effective index of refraction of the fundamental and second-order modes are shown and are calculated using ref (39). We can estimate the number of modes that can propagate along the fiber, which has a core diameter d, using the *V*-number
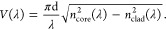
1

The number of modes that can propagate at a particular wavelength λ is then *V*^2^(λ)/2. For the excitation wavelength of 810 nm, approximately 16 modes can propagate in our LCF. For the fluorescence wavelengths of AF455 in toluene, which is peaked at 451 nm, approximately 80 modes can propagate. In the ideal case, all the light would remain in the fundamental mode because this mode experiences the lowest loss, has lower dispersion and has a Gaussian spatial profile. All these characteristics will increase the rate of 2PA.

In addition to toluene allowing broadband guidance of light in a silica fiber, this solvent also has a low absorption coefficient^40^ at the excitation and fluorescence wavelengths (0.0030 and 0.0039 cm^–1^, respectively). This is a necessary condition otherwise the long length of the fiber will add little to no benefit since all the light would be absorbed after a short distance. For comparison, water has an absorption coefficient that is about 1 order of magnitude larger at 810 nm (0.0209 cm^–1^).^40^

## Methods

**Figure 2:**
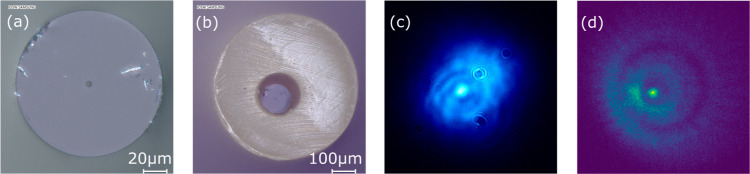
Digital microscope images of one end of (a) the fiber and (b) the fiber inside of the tubing sleeve. (c) sCMOS image of the 810 nm excitation laser guided through the fiber. (d) EMCCD image of the fluorescence from AF455 guided out of the fiber. The last two images are taken in the image plane of the fiber face. Some features of the fiber and tubing sleeve are visible in both images as described in the Supporting Information.

To prepare the fiber, we first cut clean facets on both ends to minimize light-coupling losses. We use a custom-built coil heater to remove about 20 mm of the polyamide coating from each end, then we cleave the fiber ends in the regions where the coatings were removed. We inspect the ends under a digital microscope (Keyence VHX 7000) to ensure the cuts are smooth and to check for particle contamination. Typical images are shown in Figure 2a,b. The fiber ends are placed in a tubing sleeve to prevent breakage and to allow for simple attachment to the custom-built fiber adapters (similar to those used in refs (41 and 42), technical drawings provided in ref (43)).

**Figure 3:**
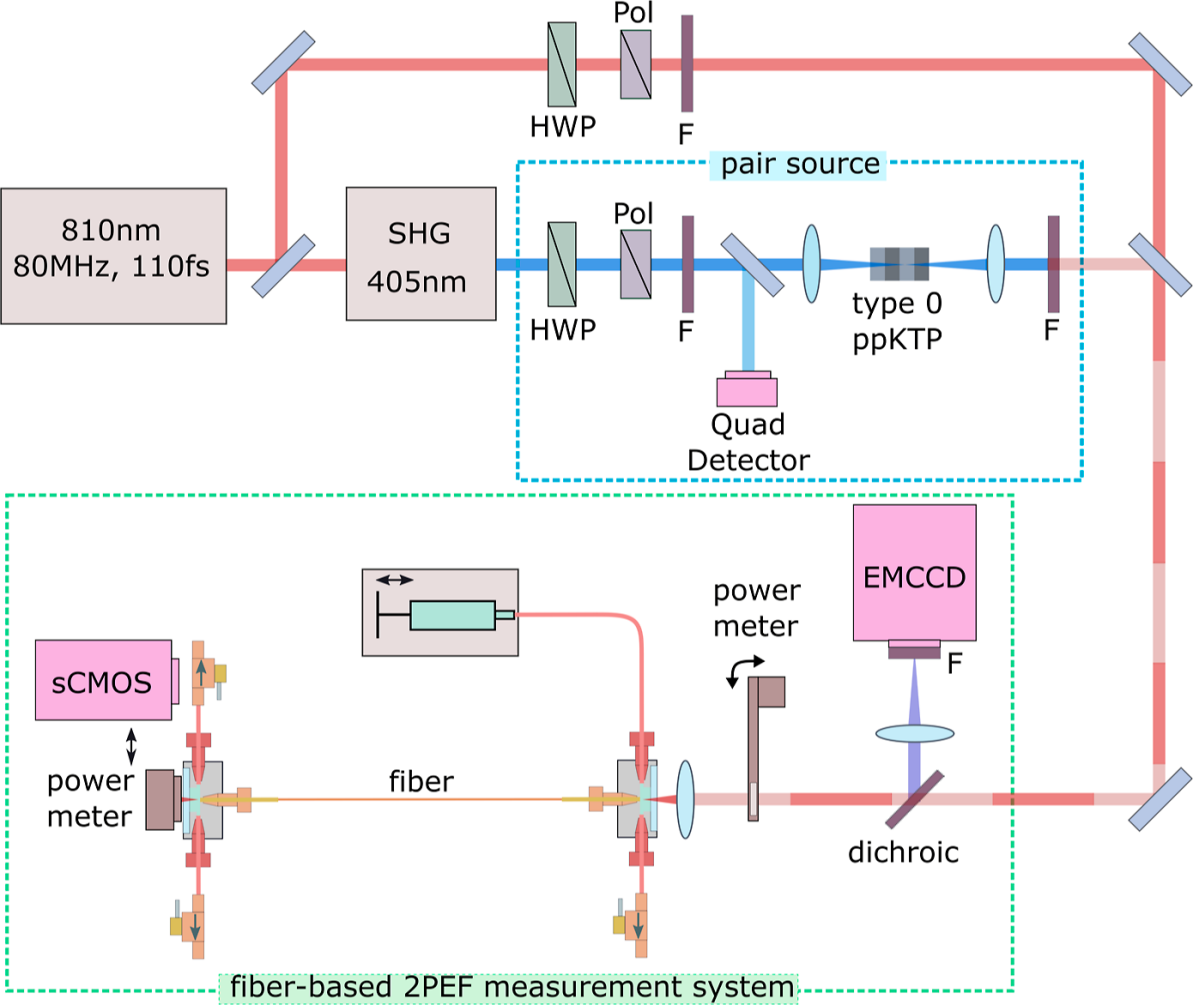
Schematic of the experimental setup. The 810 nm, 110 fs pulsed laser is split. Along one path the light is sent through a second-harmonic generation unit to frequency double the light. It is then sent through a telescope containing a type-0 ppKTP crystal used to generate photon pairs. Along the other path the beam is directed around the nonlinear crystals and is recombined with the light along the other path at a back-polished mirror. Either beam is sent through a dichroic beamsplitter and focused into the fiber held in two fiber adapters at either end. Solvent or a solution is pumped into the fiber using a syringe and syringe pump connected by PEEK tubing to one of the fiber adapters. Light transmitted through the fiber can be detected on a power meter or an sCMOS camera. A fraction of the fluorescence generated in the solution is directed out of the fiber in the direction of the dichroic beamsplitter, where it is reflected and focused onto the EMCCD camera. SHG = second-harmonic generation, HWP = half-wave plate, pol = polarizer, F = spectral filters, 2PEF = two-photon excited fluorescence.

The fiber and tubing sleeves are secured into the fiber adapters as shown in Figure 3. One fiber adapter is connected to a syringe placed into a syringe pump that is used to fill the fiber. Both fiber adapters are connected to valves that serve as drainage ports. The fiber adapters are fitted for fused silica optical windows, which are sealed onto the front for coupling light into and out of fiber. These windows also serve as viewports to check whether fluid has flowed through the fiber.

The optical and microfluidic setup is illustrated in Figure 3. Here we give a brief overview of the setup. A complete list of the components is given in ref (43). A tunable femtosecond pulsed laser operated at 810 nm (110 fs pulses, ≈9 nm bandwidth) is used to generate SPDC photons for E2PA measurements and its direct output is used for C2PA measurements. For photon pair generation, the output is first directed through a second harmonic generation unit to produce 405 nm light (≈3 nm bandwidth). The average power is controlled using a half-wave plate and a polarizer. Spectral filters are used to remove any remaining 810 nm light or unwanted harmonics. A small portion of this beam is picked off by a beam sampler to monitor the power and beam pointing stability of the laser on a quadrant (quad) detector. The blue light is focused (*f* = 300 mm lens) into a 10 mm-long type-0 periodically poled potassium titanyl phosphate (ppKTP) crystal to generate photon pairs centered at 810 nm. The crystal is temperature controlled at 40.00 ± 0.01 °C. The photon pairs are collimated with a 50 mm lens and any remaining pump light is filtered out.

In our previous report,^16^ we found that this source has a spectral bandwidth of approximately 75 nm full width at half-maximum (fwhm) under these operating conditions. We measure that this source produces 8.25 × 10^9^ photons s^–1^ mW^–1^ using a scientific Complementary Metal–Oxide–Semiconductor (sCMOS) camera placed directly after the crystal and collimating lens. This corresponds to a mean photon number of ≈5000 photons pulse^–1^ at our maximum pump power of 49.2 mW. We can estimate the number of SPDC spatial modes based on the fraction of the total photon rate that is collected into the few-mode fiber and accounting for losses due to fiber coupling, and absorption and scattering in toluene as discussed in the Supporting Information. This yields ≈740 spatial modes, which serves as a lower bound since up to 16 spatial modes can propagate in our fiber. Using this estimate, we find that we generate ≈6.8 photons pulse^–1^ spatial mode^–1^. The entanglement time of the SPDC is estimated using a discrete Fourier transform of the joint spectral amplitude, which is estimated using the measured joint spectral intensity (shown in ref (16)) and a phase estimated using the calculated group delay dispersion accumulated by the SPDC between the center of the crystal and its position along the fiber length. The broadening of the entanglement time across the length of the fiber is taken into account in our calculations (eq S26 of Supporting Information). At the entrance of the fiber the entanglement time is 1070 fs. The entanglement area was not measured, but we estimate it to be in the range of 2.1–18 μm^2^. The lower bound is set by a diffraction limited spot size (see ref (16)). The upper bound is set by the spot size of the fundamental mode of the fiber.

For the C2PA measurements, the pulsed laser is directed around the nonlinear crystals and through a half-wave plate, polarizing beamsplitter and neutral density filters for control over the power. The light from both sources is recombined at a back-polished mirror. Either source is directed into the fiber-based two-photon excited fluorescence (2PEF) measurement system. First the light propagates through a dichroic beamsplitter and is focused (*f* = 10 mm) into the 5 μm-diameter-core fiber. Light that is transmitted through the 37 cm-long fiber can be detected on a power meter or imaged on an sCMOS camera as shown in Figure 2c. The power before the fiber can be measured with a power meter that flips into the beam path, which doubles as a beam block for background measurements. Any fluorescence generated in the core of the fiber and guided out in the direction opposite of the 810 nm excitation beam is reflected at a dichroic beamsplitter and focused onto an electron-multiplying charge-coupled device (EMCCD) camera as shown in Figure 2d. Spectral filters are used to remove scattered 810 nm light.

Two fibers are used for the experiments: fiber 1 is used for the two lower sample concentration C2PA measurements (experiments 1 and 2) and fiber 2 is used for the highest sample concentration C2PA measurement (experiment 3) and for the E2PA measurement. The summed fiber scattering and toluene absorption coefficients are measured using long exposure images taken by a smartphone camera. The integrated intensity along the length of the fibers is fit to an exponential decay function as shown in Figure S1. The scattering coefficient at 810 nm is determined to be negligible whereas the absorption coefficient is equated to that found in literature.^40^ At 458 nm (near the fluorescence maximum) the summed coefficient is measured to be 0.093 cm^–1^ for fiber 1 and 0.034 cm^–1^ for fiber 2.

The measured transmission efficiency of the laser through the fiber was about 40%, 43% and 47% for experiments 1, 2, and 3 respectively; losses due to coupling, absorption in toluene (11%) and scattering are all accounted for in this efficiency. This high transmission is consistent with the light primarily occupying the fundamental mode. The average laser transmission efficiency serves as a best estimate for the transmission efficiency of the fundamental mode of SPDC (43%). If we consider all spatial modes, including those that are irrelevant in the estimation of E2PA signals since they are not coupled into fiber, we can estimate an effective transmission efficiency of 0.07%. The huge difference in transmission efficiency between a single mode and ≈740 modes illustrates that filtering the SPDC to a small number of modes may give the same result as the experiment we present. The fiber has a lower retention of light in higher-order modes and thus it effectively acts like a single-mode filter. Details on the definition and measurement of transmission efficiency for both laser and SPDC are given in the Supporting Information.

To estimate the efficiency of coupling one photon into fiber given that its spatially correlated partner photon is coupled into fiber, we estimate an effective Klyshko^44^ efficiency using SPDCalc.^45^ SPDCalc uses various parameters of our pump beam, crystal, lenses and fiber, to calculate the overlap integral of three Gaussian modes—one for signal photons, one for idler photons, and one for the collected single mode in fiber—along the length of the crystal. This yields η_*K*_^′^ = 0.94. This differs from a measured Klyshko efficiency, η_*K*_, because it does not account for any single photon loss between photon pair generation and collection into fiber. We multiply this value by the measured free-space transmission efficiency and the single-mode coupling efficiency to estimate η_*K*_ = 0.25. This value is used to estimate that 25% of photons coupled into fiber are part of an intact pair. See eq S5 on Klyshko efficiency in the Supporting Information for more details. The sample AF455 is chosen for our measurements due to its large C2PA cross-section at 810 nm and its solubility in toluene. The “AF455” fluorophore is provided by the Air Force Research Laboratory.^46,47^ The toluene used to prepare the sample has a purity of ≥99.98%. The concentrations were calculated from the one-photon absorption spectra by use of a UV–Vis–NIR spectrophotometer (Agilent Cary 5000 Scan). The concentration of the sample is monitored over the course of a measurement and found to vary by ≤ 12%

## Results and Discussion

### Classical Two-Photon Absorption Measurements

Classical two-photon excited fluorescence (C2PEF) data sets were gathered for three different concentrations of AF455 in toluene. Data series were acquired at a variety of laser powers until the uncertainty, set by the Allan deviation, reached a value at least 20% smaller than the photon rate derived from the measurement. For high excitation powers, the acquisition time was as little as 5 min, which produced uncertainty values of less than 1%. Our longest data series was acquired over a 13 h period, which lowered the uncertainty to the point where the previously indistinguishable signal could be discerned.

**Figure 4:**
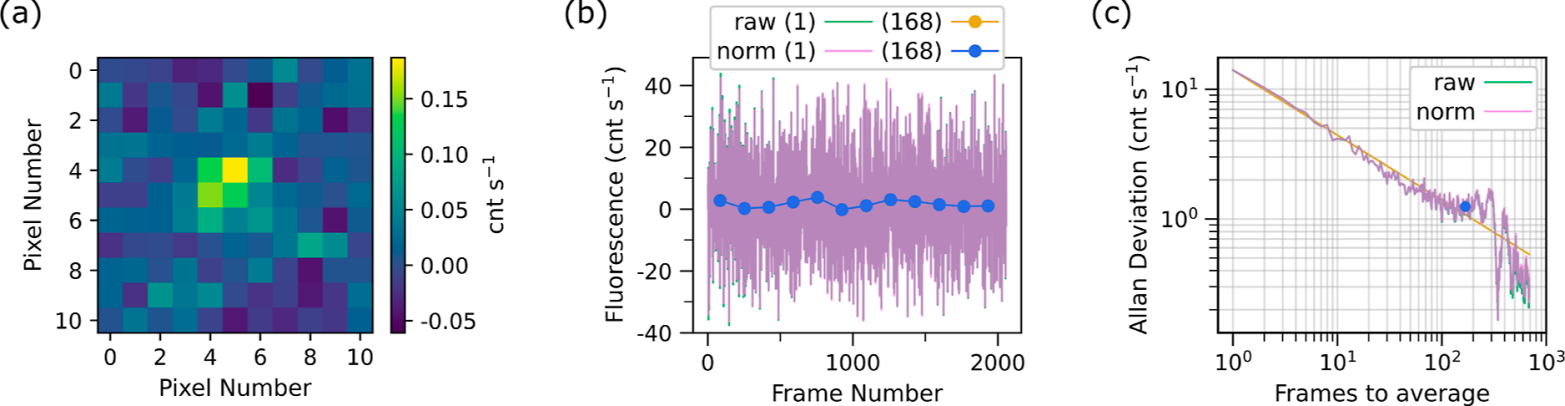
Data series for 1.75 nW average laser excitation power used with the 2.30 mM sample. (a) The average background-subtracted image is shown for the 11 × 11 superpixel region of interest. (b) The fluorescence rate calculated from each frame’s background-subtracted image is plotted in teal for the raw data or pink for the normalized (norm) data as a function of frame number. The overlap of the raw and normalized data is indicated by the purple trace. Averaging in portions of 168 frames is shown in gold for the raw data or blue for the normalized data. Averaging the normalized data gives a photon rate of 1.6 counts per second (cnt s^–1^). The normalized data for both the individual and averaged data follows the raw data closely and thus nearly perfectly overlaps the raw data on the plot. This trend indicates a fairly stable laser. (c) The Allan deviation of the photon rate is plotted as a function of number of frames used to average. The teal data shows the Allan deviation of the raw data and the pink shows that for the normalized data. The overlap of the two traces is indicated in purple. A gold  (where *N* is the number of frames) line is used to guide the eye. The Allan deviation value used in the analysis is indicated by a blue data point, corresponding to averaging portions of 168 frames with an Allan deviation of 1.3 cnt s^–1^. This data series consists of 2058 frames, which required about 13 h to collect.

An example data series is shown in Figure 4 for the lowest average excitation power (*W*_0_ in eq S2 of the Supporting Information) of 1.75 nW. This data series was acquired over ≈13 h during experiment 3. In Figure 4a, the background-subtracted image, which is averaged over the duration of the data series, is shown for the 11 × 11 superpixel (24 × 24 pixels per superpixel) region of interest. Near the center of the image a resolved bright spot shows the signal. Without binning, and at higher excitation powers, this image looks similar to Figure 2d.

In Figure 4b the fluorescence rate extracted from each frame’s background-subtracted image is plotted as a function of frame number for both raw (teal) and normalized (pink) data. The normalized data accounts for any changes in laser excitation power (see Supporting Information). In Figure 4c the Allan deviation of the fluorescence rate is plotted for different numbers of frames (*N*) used for averaging for both raw (teal) and normalized (pink) data. For both Figure 4b,c, the overlap of the raw and normalized data is indicated by a purple trace. The Allan deviation should follow a  line in the absence of noise sources at that combined frame rate, thus this gold line is used to pick a near optimum number of frames to average. Here, 168 frames is selected, which corresponds to an Allan deviation of 1.3 cnt s^–1^. We average the data in portions of 168 frames and plot the result in Figure 4b for the raw (gold) and normalized (blue) data. These measurements have an average value of 1.6 cnt s^–1^.

**Figure 5:**
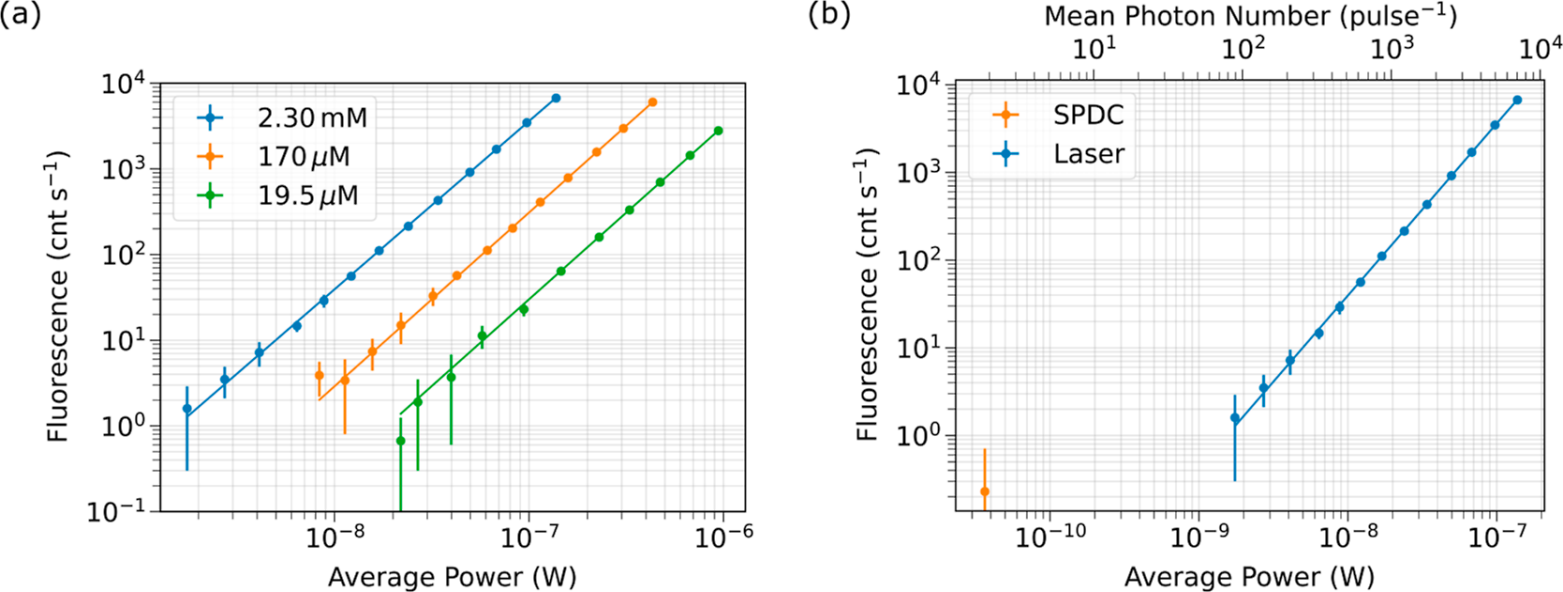
(a) The fluorescence rate measured as a function of the average laser power input to the fiber for a sample of 2.30 mM, 170 and 19.5 μM AF455 in toluene shown in blue, orange and green, respectively. The linear fits to the data sets have slopes of 1.96, 2.03, and 2.02 respectively. (b) The fluorescence rate measured as a function of the power input to the fiber for both SPDC (orange) and laser (blue) excitation for a sample of 2.30 μM AF455 in toluene. The SPDC measurement is performed at a power 48-fold lower than the minimum laser power and is indistinguishable from zero.

The results of our fluorescence measurements for three concentrations of AF455 in toluene are plotted in Figure 5a, which shows the fluorescence count rate as a function of average excitation power. The fits to the data sets are linear regressions performed on a log–log scale. The slopes of the fits are all within 0.05 of 2.00, which confirms the two-photon origin of the signals. The lowest data point (acquired using the data shown in Figure 4) is measured for the highest concentration at a power of 1.75 nW. The signals shown here are compared with our model using measured and calculated parameters in Figure S2 and show qualitative agreement.

For all three AF455 concentrations, we find that C2PA can be measured at excitation powers significantly lower than is typically possible with a free-space technique. Table 1 compares the parameters used in this experiment to our previous experiment performed in free space.^16^ In our previous report, for a sample of 1.10 mM AF455 in toluene, C2PA can be measured down to 79 nW. In this work, we show a 45-fold improvement. The advantage of this platform is ultimately due to the capability to generate high photon fluxes with low average powers by focusing the light to a very small spot size and maintaining it over the length of the fiber. The peak photon flux at our minimum excitation power of 1.75 nW is 1.1 × 10^22^ photons cm^–2^ s^–1^; in the earlier technique at the minimum power of 79 nW, the peak photon flux was 1.3 × 10^21^ photons cm^–2^ s^–1^. Thus, an 8.5-fold higher photon flux was achieved with a 45-fold lower power.

**Table 1:** Comparison of Fiber to Free-Space AF455 Measurements

parameter	unit	fiber (this work)	free space (ref (16))
concentration	mM	2.30	1.10
min. laser power (flux)	nW (cm^–2^ s^–1^)	1.75 (1.1 × 10^22^)^a^	79 (1.3 × 10^21^)
C2PA cross-section	GM	390 ± 80	660 ± 180
entanglement area	μm^2^	2.1 to 18	2.1 to 13,700
entanglement time	fs	1070^a^	1620
SPDC power (flux)	pW (cm^–2^ s^–1^)	36.5 (7.7 × 10^19^)^a^	2200 (2.1 × 10^18^)
SPDC loss	%	73^a^	24
intact pairs	%	25^a^	58
E2PA cross-section UB	cm^2^	(5.8 ± 2.3) × 10^–24^	(2.1 ± 0.5) × 10^–25^
*R*^UB^		8.5	

a This value is valid at the front of the fiber (*z* = 0).

Using eq S19 of the Supporting Information, C2PA cross-sections are derived using the fits to each of the three concentration data sets. We derive values of (570 ± 190) GM, (340 ± 120) GM and (250 ± 80) GM for the 2.30 mM, 170 and 19.5 μM samples, respectively. The average of these is (390 ± 80) GM which is in line with the cross-sections reported for AF455.^16,48^

### Entangled Two-Photon Absorption Measurement

**Figure 6:**
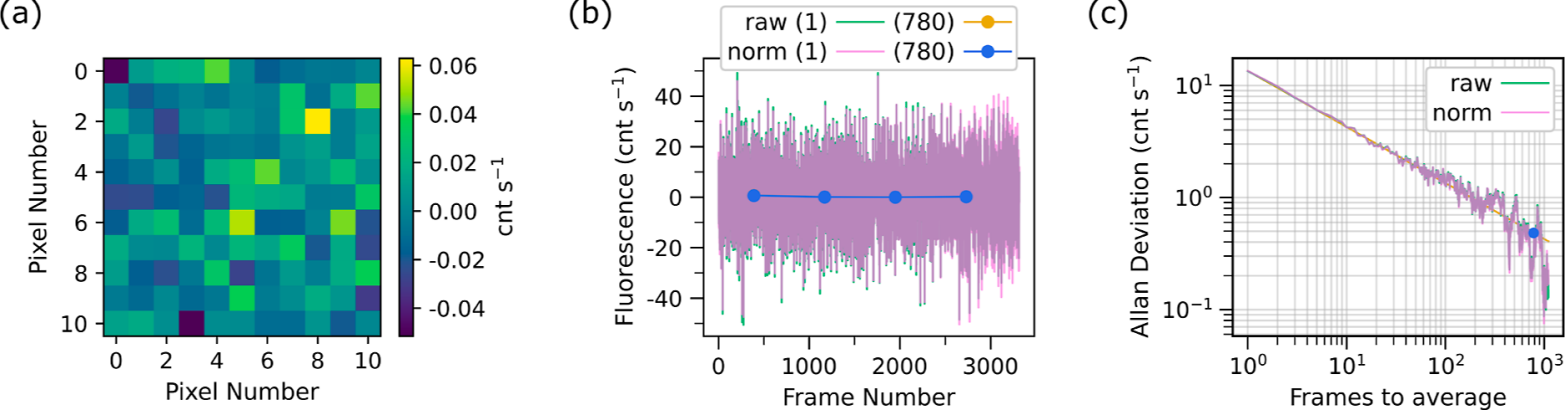
Data series for SPDC excitation of the 2.30 mM sample. (a) The average background-subtracted image is shown for the 11 × 11 superpixel region of interest. (b) The fluorescence rate calculated from each frame’s background-subtracted image is plotted in teal for the raw data or pink for the normalized (norm) data as a function of frame number. The overlap of the raw and normalized data is indicated by a purple trace. Averaging in portions of 780 frames is shown in gold for the raw data or blue for the normalized data. Averaging the raw and normalized data gives a photon rate of 0.23 and 0.22 cnt s^–1^ respectively. The normalized data for both the individual and averaged data follows the raw data closely and thus nearly perfectly overlaps the raw data on the plot. This trend indicates a fairly stable laser. (c) The Allan deviation of the photon rate is plotted as a function of number of frames used to average. The teal data shows the Allan deviation of the raw data and the pink shows that for the normalized data. A  (where *N* is the number of frames) line is used to guide the eye and is shown in gold. The Allan deviation value used in the analysis is indicated by a blue data point, corresponding to averaging portions of 780 frames with an Allan deviation of 0.48 cnt s^–1^ for both raw and normalized data. This data series consists of 3316 frames, which required about 24 h to collect.

The SPDC source was used exclusively with the 2.30 mM sample. Using the high concentration sample maximizes the likelihood of measuring entangled two-photon excited fluorescence (E2PEF). The results from the SPDC data series are shown in Figure 6. For this measurement the pump power was nearly maximum for an average of 49.2 mW, which generates 4.06 × 10^11^ photons s^–1^. A small number of modes of SPDC are effectively coupled into fiber, reducing the photon rate to 1.49 × 10^8^ photons s^–1^. The average background-subtracted image is shown in Figure 6a, where there is no discernible bright spot due to a signal. In Figure 6c, the Allan deviation is plotted, which is used to determine a near optimal averaging of 780 frames. This averaging corresponds to an uncertainty of 0.48 cnt s^–1^ for both the raw (teal) and normalized (pink) data. In Figure 6b, the fluorescence rate from each frame is plotted as well as the averaged (in 780 frame portions) fluorescence rate. The average fluorescence rate for the raw (gold) data is (0.23 ± 0.48) cnt s^–1^ and for the normalized (blue) data is (0.22 ± 0.48) cnt s^–1^. The magnitude of the signal is not significantly different from zero, and therefore we conclude that there is no resolvable signal.

Although E2PEF was indistinguishable from zero, there are numerous conclusions to be drawn from this measurement. The C2PEF data set for the 2.30 mM sample is plotted alongside our E2PEF measurement in Figure 5b. Here we show measured fluorescence count rates as a function of the average power of either the SPDC (orange) or laser (blue) source. For the E2PEF measurement, we couple about 36.5 pW (≈1.9 photons pulse^–1^) of SPDC into the fiber. This plot demonstrates the difference between the SPDC power and the minimum laser power, which are only 48-fold apart.

We can compare these E2PA results with those done in free space (Table 1). Notably, the power of SPDC at the sample is 60-fold lower in the present experiment. This is because the free-space experiment uses all the photons from ≈740 spatial modes, whereas the fiber acts as a few-mode filter. Despite this lower power, the peak photon flux is 7.7 × 10^19^ photons cm^–2^ s^–1^. In the free-space studies the SPDC peak photon flux was 2.1 × 10^18^ photons cm^–2^ s^–1^, which is 37-fold lower. It should be noted that some of the advantage of the higher SPDC photon flux is hindered by the lower fraction of intact photon pairs propagating in the fiber, which is 25% for fiber and 58% for free space. The intact pair rate is accounted for using the estimated Klyshko efficiency in the calculations in the Supporting Information.

Using eq S31 in the Supporting Information, we set an upper bound on the E2PA cross-section of this sample at (5.8 ± 2.3) × 10^–24^ cm^2^. This upper bound is for an entanglement time of 1070 fs and an entanglement area in the range 2.1–18 μm^2^. This entanglement time is an estimated value for the SPDC at the entrance of the fiber adapter. The broadening of the entanglement time in the fiber is among numerous parameters accounted for in the calculation of the upper bound (see Supporting Information).

We can compare the upper bound set here to the upper bound set in ref (16) for the same sample as shown in Table 1. Since the E2PA cross-section is known to scale with parameters of the excitation beam that varied between the two experiments, we cannot compare the two numbers alone. For instance, in an experiment where the SPDC beam is tightly focused at the sample, the entanglement area typically decreases proportionally, leading to a larger E2PA cross-section compared to experiments with gentler focusing. Similary, if the SPDC is sent through dispersive elements, the temporal separation between photons in a pair broadens—especially for SPDC sources with broad bandwidths—resulting in an increased entanglement time and a reduced E2PA cross-section. To properly compare the upper bounds, we use the probabilistic model (see for example ref (16)) and arrive at the ratio *R*^U^^B^
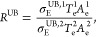
2where *T*_e_ and *A*_e_ are the entanglement time and area, 1 indicates the current experiment, and 2 indicates the previous experiment.

Although we do not know the exact entanglement areas of the SPDC used in either experiment, the same ppKTP crystal, pump laser and pump focusing conditions were used in both experiments. Assuming that the spatial correlations of the SPDC are maintained as it propagates through the optical system, the entanglement area should scale with the beam size at the image plane of the crystal. Since the optical system is designed to image the crystal into the sample, we can use this logic to estimate the ratio of the entanglement areas in the previous experiment to that in the current experiment as the ratio of the respective beam sizes of the SPDC at the sample position. For a fair comparison, we use the beam size of all ≈740 spatial modes at the focus of the lens used for fiber coupling, which is 6350 μm^2^. In free space, the beam size at its focus in the sample was 13,700 μm^2^. Plugging in the numbers we arrive at *R*^UB^ of 8.5. This result indicates that the current experiment sets an upper bound that is 8.5-fold larger than the previous experiment, and is thus 8.5-fold less stringent.

## Conclusions

We presented a toluene-filled fiber platform for two-photon excited fluorescence measurements. We used this platform to measure C2PA of various concentrations of the sample AF455. The results of the C2PA measurements show that 2PA can be measured at extremely low powers (1.75 nW) using an excitation volume of only 7.3 nL. This power is 45-fold lower than we achieved previously in free-space under similar conditions (ref (16)). Furthermore, we derived a C2PA cross-section for AF455 of (390 ± 80) GM. These results emphasize the advantages of liquid-core fiber platforms for sensing applications where one could instead use higher powers to sense very low concentrations of analytes.

With photon pair excitation, we saw no evidence of a signal, and set an upper bound on the E2PA cross-section of AF455. Our calculation of *R*^UB^, which uses σ_*E*_^UB^ to account for many experimental factors such as loss and the broadening of the entanglement time, shows that the bound we set on the E2PA cross-section is 8.5-fold larger than that set in ref (16). Thus, this technique does not provide a higher E2PA sensitivity compared to the free-space technique. However, since the SPDC loss reduces the upper bound in a quadratic manner, efforts to decrease the loss would greatly improve the sensitivity of this technique. This work furthers the growing body of research that has presented null results when attempting to measure entangled two-photon absorption (refs (16, 22–32)).

## Supporting Information Available

Additional information on experimental characterization, data acquisition and data analysis (PDF)

## References

[ref1] Dallas T.; Dasgupta P. K. Light at the end of the tunnel: recent analytical applications of liquid-core waveguides. Trends Anal. Chem. 2004, 23, 385–392. 10.1016/S0165-9936(04)00522-9.

[ref2] Khoo I. C.; Diaz A.; Ding J. Nonlinear-absorbing fiber array for large-dynamic-range optical limiting application against intense short laser pulses. J. Opt. Soc. Am. B 2004, 21, 1234–1240. 10.1364/JOSAB.21.001234.

[ref3] Calcerrada M.; García-Ruiz C.; González-Herráez M. Chemical and biochemical sensing applications of microstructured optical fiber-based systems. Laser Photonics Rev. 2015, 9, 604–627. 10.1002/lpor.201500045.

[ref4] Gerosa R. M.; Sudirman A.; Menezes L. d. S.; Margulis W.; de Matos C. J. S.; de Matos C. J. S. All-fiber high repetition rate microfluidic dye laser. Optica 2015, 2, 186–193. 10.1364/optica.2.000186.

[ref5] Scheibinger R.; Lüpken N. M.; Chemnitz M.; Schaarschmidt K.; Kobelke J.; Fallnich C.; Schmidt M. A. Higher-order mode supercontinuum generation in dispersion-engineered liquid-core fibers. Sci. Rep. 2021, 11, 5270 10.1038/s41598-021-84397-1. 33674632 PMC7935952

[ref6] Bhagwat A. R.; Gaeta A. L. Nonlinear optics in hollow-core photonic bandgap fibers. Opt. Express 2008, 16, 5035–5047. 10.1364/OE.16.005035. 18542604

[ref7] Chemnitz M.; Junaid S.; Schmidt M. A. Liquid-Core Optical Fibers—A Dynamic Platform for Nonlinear Photonics. Laser Photonics Rev. 2023, 17, 2300126 10.1002/lpor.202300126.

[ref8] He G. S.; Bhawalkar J. D.; Zhao C. F.; Park C.-K.; Prasad P. N. Two-photon-pumped cavity lasing in a dye-solution-filled hollow-fiber system. Opt. Lett. 1995, 20, 2393–2395. 10.1364/OL.20.002393. 19865230

[ref9] Khoo I. C.; Wood M. V.; Lee M.; Guenther B. D. Nonlinear liquid-crystal fiber structures for passive optical limiting of short laser pulses. Opt. Lett. 1996, 21, 1625–1627. 10.1364/OL.21.001625. 19881747

[ref10] He G. S.; Yuan L.; Bhawalkar J. D.; Prasad P. N. Optical limiting, pulse reshaping, and stabilization with a nonlinear absorptive fiber system. Appl. Opt. 1997, 36, 3387–3392. 10.1364/AO.36.003387. 18253353

[ref11] Khoo I. C.; Wood M. V.; Guenther B. D.; Shih M.-Y.; Chen P. H. Nonlinear absorption and optical limiting of laser pulses in a liquid-cored fiber array. J. Opt. Soc. Am. B 1998, 15, 1533–1540. 10.1364/JOSAB.15.001533.

[ref12] Williams G. O. S.; Euser T. G.; Arlt J.; Russell P. S.; Jones A. C. Taking Two-Photon Excitation to Exceptional Path-Lengths in Photonic Crystal Fiber. ACS Photonics 2014, 1, 790–793. 10.1021/ph5002236.

[ref13] Saha K.; Venkataraman V.; Londero P.; Gaeta A. L. Enhanced two-photon absorption in a hollow-core photonic-band-gap fiber. Phys. Rev. A 2011, 83, 033833 10.1103/PhysRevA.83.033833. 22181608

[ref14] Hendrickson S. M.; Lai M.; Pittman T.; Franson J. Observation of Two-Photon Absorption at Low Power Levels Using Tapered Optical Fibers in Rubidium Vapor. Phys. Rev. Lett. 2010, 105, 173602 10.1103/PhysRevLett.105.173602. 21231044

[ref15] Perrella C.; Griesser H. P.; Light P. S.; Kostecki R.; Stace T. M.; Ebendorff-Heidepriem H.; Monro T. M.; White A.; Luiten A. Demonstration of an Exposed-Core Fiber Platform for Two-Photon Rubidium Spectroscopy. Phys. Rev. Appl. 2015, 4, 014013 10.1103/PhysRevApplied.4.014013.

[ref16] Parzuchowski K. M.; Mikhaylov A.; Mazurek M. D.; Wilson R. N.; Lum D. J.; Gerrits T.; Camp C. H.; Stevens M. J.; Jimenez R.; Jimenez R. Setting Bounds on Entangled Two-Photon Absorption Cross Sections in Common Fluorophores. Phys. Rev. Appl. 2021, 15, 044012 10.1103/physrevapplied.15.044012.

[ref17] Rumi M.; Perry J. W. Two-photon absorption: an overview of measurements and principles. Adv. Opt. Photonics 2010, 2, 451–518. 10.1364/AOP.2.000451.

[ref18] Javanainen J.; Gould P. L. Linear intensity dependence of a two-photon transition rate. Phys. Rev. A 1990, 41, 5088–5091. 10.1103/PhysRevA.41.5088. 9903733

[ref19] Gea-Banacloche J. Two-photon absorption of nonclassical light. Phys. Rev. Lett. 1989, 62, 1603–1606. 10.1103/PhysRevLett.62.1603. 10039717

[ref20] Fei H.-B.; Jost B. M.; Popescu S.; Saleh B. E. A.; Teich M. C. Entanglement-Induced Two-Photon Transparency. Phys. Rev. Lett. 1997, 78, 1679–1682. 10.1103/PhysRevLett.78.1679.

[ref21] Raymer M. G.; Landes T. Theory of two-photon absorption with broadband squeezed vacuum. Phys. Rev. A 2022, 106, 013717 10.1103/PhysRevA.106.013717.

[ref22] Landes T.; Smith B. J.; Raymer M. G. Limitations in fluorescence-detected entangled two-photon-absorption experiments: Exploring the low- to high-gain squeezing regimes. Phys. Rev. A 2024, 110, 033708 10.1103/PhysRevA.110.033708.

[ref23] Mikhaylov A.; Parzuchowski K. M.; Mazurek M. D.; Lum D. J.; Gerrits T.; Camp C. H.; Stevens M. J.; Jimenez R. In A Comprehensive Experimental System for Measuring Molecular Two-Photon Absorption Using an Ultrafast Entangled Photon Pair Excitation Source. Adv. Opt. Tech. for Quantum Information, Sensing, and Metrology; SPIE, 2020. 10.1117/12.2541888

[ref24] Mazurek M. D.; Parzuchowski K. M.; Mikhaylov A.; Nam S. W.; Camp C. H.; Gerrits T.; Jimenez R.; Stevens M. J. In Bounding Entangled Two-Photon Absorption with Sensitive Transmittance Measurements. Conf. on Lasers and Electro-Optics; Optica Publishing Group, 2021.

[ref25] Landes T.; Allgaier M.; Merkouche S.; Smith B. J.; Marcus A. H.; Raymer M. G. Experimental feasibility of molecular two-photon absorption with isolated time-frequency-entangled photon pairs. Phys. Rev. Res. 2021, 3, 033154 10.1103/PhysRevResearch.3.033154.

[ref26] Mikhaylov A.; Wilson R. N.; Parzuchowski K. M.; Mazurek M. D.; Camp C. H.; Stevens M. J.; Jimenez R. Hot-Band Absorption Can Mimic Entangled Two-Photon Absorption. J. Phys. Chem. Lett. 2022, 13, 1489–1493. 10.1021/acs.jpclett.1c03751. 35129354

[ref27] Corona-Aquino S.; Calderón-Losada O.; Li-Gómez M. Y.; Cruz-Ramirez H.; Álvarez-Venicio V.; Carreón-Castro M. d. P.; de J León-Montiel R.; U’Ren A. B.; U’Ren A. B. Experimental Study of the Validity of Entangled Two-Photon Absorption Measurements in Organic Compounds. J. Phys. Chem. A 2022, 126, 2185–2195. 10.1021/acs.jpca.2c00720. 35383460

[ref28] Hickam B. P.; He M.; Harper N.; Szoke S.; Cushing S. K. Single-Photon Scattering Can Account for the Discrepancies among Entangled Two-Photon Measurement Techniques. J. Phys. Chem. Lett. 2022, 13, 4934–4940. 10.1021/acs.jpclett.2c00865. 35635002

[ref29] Triana-Arango F.; Ramos-Ortiz G.; Ramírez-Alarcón R. Spectral Considerations of Entangled Two-Photon Absorption Effects in Hong–Ou–Mandel Interference Experiments. J. Phys. Chem. A 2023, 127, 2608–2617. 10.1021/acs.jpca.2c07356. 36913489

[ref30] Gäbler T. B.; Hendra P.; Jain N.; Gräfe M. Photon Pair Source based on PPLN-Waveguides for Entangled Two-Photon Absorption. Adv. Physics Res. 2024, 3, 2300037 10.1002/apxr.202300037.

[ref31] Qian G.; Liu X.; Xu C.; Xu X.; Wang D.-W. Experimental test of the entanglement enhancement in two-photon fluorescence. Quantum Front 2024, 3, 5 10.1007/s44214-024-00052-6.

[ref32] He M.; Hickam B. P.; Harper N.; Cushing S. K. Experimental upper bounds for resonance-enhanced entangled two-photon absorption cross section of indocyanine green. J. Chem. Phys. 2024, 160, 094305 10.1063/5.0193311. 38445732

[ref33] Tabakaev D.; Djorović A.; La Volpe L.; Gaulier G.; Ghosh S.; Bonacina L.; Wolf J.-P.; Zbinden H.; Thew R. T. Spatial Properties of Entangled Two-Photon Absorption. Phys. Rev. Lett. 2022, 129, 183601 10.1103/PhysRevLett.129.183601. 36374702

[ref34] Raymer M. G.; Landes T.; Marcus A. H. Entangled two-photon absorption by atoms and molecules: A quantum optics tutorial. J. Chem. Phys. 2021, 155, 081501 10.1063/5.0049338. 34470351

[ref35] Landes T.; Raymer M. G.; Allgaier M.; Merkouche S.; Smith B. J.; Marcus A. H. Quantifying the enhancement of two-photon absorption due to spectral-temporal entanglement. Opt. Express 2021, 29, 20022–20033. 10.1364/OE.422544. 34266101

[ref36] Drago C.; Sipe J. E. Aspects of two-photon absorption of squeezed light: The continuous-wave limit. Phys. Rev. A 2022, 106, 023115 10.1103/PhysRevA.106.023115.

[ref37] Moutzouris K.; Papamichael M.; Betsis S. C.; Stavrakas I.; Hloupis G.; Triantis D. Refractive, dispersive and thermo-optic properties of twelve organic solvents in the visible and near-infrared. Appl. Phys. B: Laser Opt. 2014, 116, 617–622. 10.1007/s00340-013-5744-3.

[ref38] Malitson I. H. Interspecimen Comparison of the Refractive Index of Fused Silica. J. Opt. Soc. Am. 1965, 55, 1205–1209. 10.1364/JOSA.55.001205.

[ref39] Fini J. M. Microstructure fibres for optical sensing in gases and liquids. Meas. Sci. Technol. 2004, 15, 1120 10.1088/0957-0233/15/6/011.

[ref40] Kedenburg S.; Vieweg M.; Gissibl T.; Giessen H. Linear refractive index and absorption measurements of nonlinear optical liquids in the visible and near-infrared spectral region. Opt. Mater. Express 2012, 2, 1588–1611. 10.1364/OME.2.001588.

[ref41] Frosch T.; Yan D.; Popp J. Ultrasensitive Fiber Enhanced UV Resonance Raman Sensing of Drugs. Anal. Chem. 2013, 85, 6264–6271. 10.1021/ac400365f. 23758275

[ref42] Yan D.; Popp J.; Pletz M. W.; Frosch T. Highly Sensitive Broadband Raman Sensing of Antibiotics in Step-Index Hollow-Core Photonic Crystal Fibers. ACS Photonics 2017, 4, 138–145. 10.1021/acsphotonics.6b00688.

[ref43] Parzuchowski K. M. Setting Experimental Bounds on Entangled Two-Photon Absorption Cross Sections. PhD Thesis; University of Colorado: Boulder, Boulder, CO, 2023.

[ref44] Klyshko D. N. Use of two-photon light for absolute calibration of photoelectric detectors. Sov. J. Quantum Electron. 1980, 10, 1112–1116. 10.1070/QE1980v010n09ABEH010660.

[ref45] Shalm L. K. SPDCalc application. https://app.spdcalc.org/, (accessed) 13–01 2025.

[ref46] Kannan R.; He G. S.; Lin T.-C.; Prasad P. N.; Vaia R. A.; Tan L.-S. Toward Highly Active Two-Photon Absorbing Liquids. Synthesis and Characterization of 1,3,5-Triazine-Based Octupolar Molecules. Chem. Mater. 2004, 16, 185–194. 10.1021/cm034358g.

[ref47] Rogers J. E.; Slagle J. E.; McLean D. G.; Sutherland R. L.; Sankaran B.; Kannan R.; Tan L.-S.; Fleitz P. A. Understanding the One-Photon Photophysical Properties of a Two-Photon Absorbing Chromophore. J. Phys. Chem. A 2004, 108, 5514–5520. 10.1021/jp048961d.

[ref48] de Reguardati S.; Pahapill J.; Mikhailov A.; Stepanenko Y.; Rebane A. High-accuracy reference standards for two-photon absorption in the 680–1050 nm wavelength range. Opt. Express 2016, 24, 9053–9066. 10.1364/oe.24.009053. 27137334 PMC5025204

