# Supplementary material for: A Liquid-Core Fiber Platform for Classical and Entangled Two-Photon Absorption Measurements
Source: ACS Photonics. 2025 Mar 7;12(3):1470–9. doi: 10.1021/acsphotonics.4c02076 (PMC11926961; doi:10.1021/acsphotonics.4c02076)
Supplement: Supplementary file 1 — ph4c02076_si_001.pdf [file ph4c02076_si_001.pdf]

Supporting Information

A Liquid-Core Fiber Platform for Classical and Entangled Two-Photon Absorption Measurements

Kristen M. Parzuchowski,[*,†,‡,¶] Michael D. Mazurek,[¶,‡] Charles H. Camp Jr.,[§] Martin J. Stevens,[∥] and Ralph Jimenez[*,†,⊥]

†*JILA, University of Colorado Boulder, Boulder, Colorado 80309, USA*
‡*Department of Physics, University of Colorado Boulder, Boulder, Colorado 80309, USA*
¶*Associate of the National Institute of Standards and Technology, Boulder, Colorado 80305, USA*
§*National Institute of Standards and Technology, Gaithersburg, Maryland 20899, USA*
∥*National Institute of Standards and Technology, Boulder, Colorado 80305, USA*
⊥*Department of Chemistry, University of Colorado Boulder, Boulder, Colorado 80309, USA*

E-mail: kristen.parzuchowski@nist.gov; rjimenez@jila.colorado.edu

Experimental Characterization

In this section we discuss the experimental characterizations we performed to derive C2PA cross-sections using Eq. (19) and an E2PA cross-section upper bound using Eq. (31). First, we discuss the transmission efficiency and propagation losses of the fiber. Next, we discuss the considerations needed to switch from single-mode laser light to multimode SPDC. We characterize the number of spatial modes and the Klyshko efficiency. Afterwards, we discuss images of the excitation and fluorescence light at the fiber face for qualitative identification of the mode content of the fiber. Finally, we discuss our characterization of the setup's dispersion.

Before C2PA experiments we maximize the transmission of the 810 nm laser light through the fiber. We measure the power before and after the fiber using power meters, and the ratio of the power transmitted out W_{out} (W) to the incident power W_{in} (W) is what we call our transmission efficiency,

$$\begin{aligned}\eta_T &= \frac{W_{\text{out}}}{W_{\text{in}}} \\ &= \eta_C \eta_A(\lambda = \lambda_e, z = l)\eta_S(\lambda = \lambda_e, z = l).\end{aligned} \quad (1)$$

This efficiency is a product of coupling (η_C), absorption (η_A) and scattering (η_S) efficiencies. The latter two efficiencies are evaluated at the excitation wavelength (λ_e = 810 nm) and for the entire length of the fiber l (cm). The best alignment resulted in $\eta_T > 55\%$. However, during our measurements $\eta_T \approx 43\%$ but varied from data series to data series. This value for η_T serves as a best estimate for the transmission efficiency for any aligned single spatial mode source at the same frequency, including a single mode of SPDC. Similarly, the values of the components of η_T: η_C, η_A and η_S, derived using this measurement and the scattering measurements as described below, also serve as a best estimate of these parameters for any single mode of the same frequency.

We characterize the loss of light along the

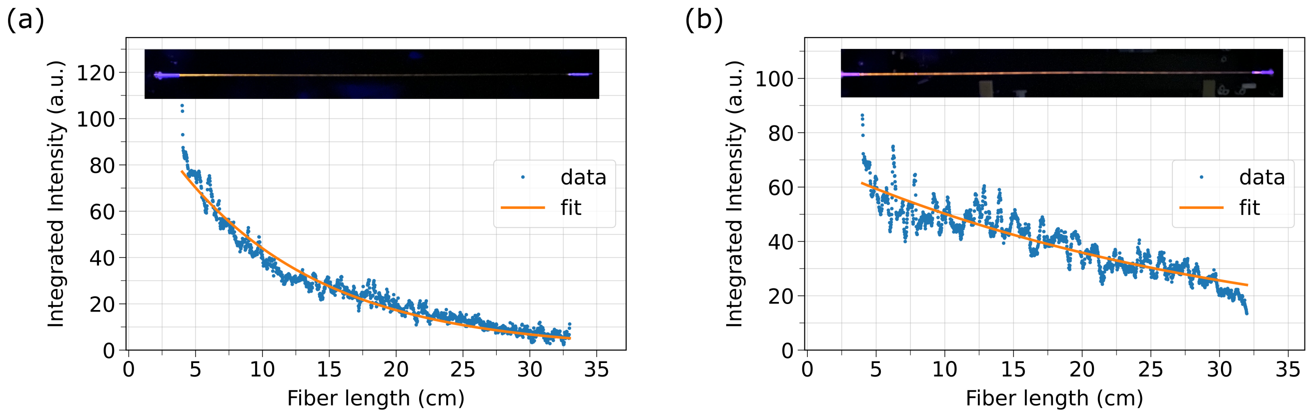

Figure S1: Images of the scatter from the alignment laser (inset) with corresponding plots of the integrated intensity (blue) of the scatter as a function of fiber length for (a) fiber 1 and (b) fiber 2. The intensity of the scatter is higher at the ends due to secondary reflections off the fiber tubing sleeves and fittings. These regions are removed from the plotted data. The fiber is not visible inside of the fittings and fiber adapters. The integrated intensity is fit (orange) to an exponential decay.

length z of the fiber by imaging the scattering. The intensity of the scattering is proportional to the power $W(z)$ (W), and thus should exponentially decay according to

$$W(z) = W_0 \times \mathrm{Exp}\left(-(a_{\mathrm{sol}}(\lambda) + \epsilon_{\mathrm{sam}}(\lambda)c + \mu(\lambda))z\right), \tag{2}$$

where W_0 (W) is the average power at $z = 0$, λ (nm) is the wavelength of the light, $a_{\mathrm{sol}}(\lambda)$ (cm^{-1}) is the absorption coefficient of the solvent, $\epsilon_{\mathrm{sam}}(\lambda)$ ($\mathrm{M}^{-1}\,\mathrm{cm}^{-1}$) is the extinction coefficient of the sample, c (M) is the concentration of the sample and $\mu(\lambda)$ (cm^{-1}) is the scattering coefficient of the fiber. The parameter W_0 is related to the power measured before the fiber W_{in} (W) by $W_0 = \eta_C W_{\mathrm{in}}$. The power measured at the output of the fiber W_{out} is a good estimate for $W(l)$. The exponential terms related to absorption of the light are $\eta_A(\lambda, z)$ and to scattering of the light is $\eta_S(\lambda, z)$, thus we can rewrite Eq. (2) as

$$W(z) = W_{\mathrm{in}}\eta_C\eta_A(\lambda, z)\eta_S(\lambda, z). \tag{3}$$

With only the solvent in the fiber ($c = 0$), we send a 458 nm (near the peak of our sample's emission spectrum) alignment laser through the fiber and image the scattering on a smart phone camera using long exposure settings as shown in Fig. S1. The image is integrated along the width of the fiber (vertically) to find an integrated intensity as a function of fiber length. The intensity is fit to the exponential in Eq. (2) to derive $a_{\mathrm{sol}}(458\,\mathrm{nm}) + \mu(458\,\mathrm{nm}) = 0.093\ \mathrm{cm}^{-1}$ for fiber 1 (Fig. S1(a)) and $a_{\mathrm{sol}}(458\,\mathrm{nm}) + \mu(458\,\mathrm{nm}) = 0.034\ \mathrm{cm}^{-1}$ for fiber 2 (Fig. S1(b)). For 458 nm fluorescence, traveling the entire length of the fiber corresponds to a loss of 96.8% or 70.6% of the light for fibers 1 and 2 respectively. We take the same measurement with 750 nm (a visible wavelength close to our excitation wavelength) light and observe no evidence of decay of the scatter along the length of the fiber. We estimate that $a_{\mathrm{sol}}(750\,\mathrm{nm}) + \mu(750\,\mathrm{nm})$ is not large enough to measure this way. As mentioned in the main text, the values of $a_{\mathrm{sol}}(\lambda)$ are known. From literature,[1] $a_{\mathrm{sol}}(750\,\mathrm{nm}) = 0.0036\ \mathrm{cm}^{-1}$ and $a_{\mathrm{sol}}(810\,\mathrm{nm}) = 0.0030\ \mathrm{cm}^{-1}$. If we set $\mu(750\,\mathrm{nm}) = \mu(810\,\mathrm{nm}) = 0$, we find that these absorption coefficients correspond to a loss of 12.5% and 10.5%, respectively, of the light along the length of the fiber. Considering with our best alignment $\eta_T > 55\%$, and that some fraction of that light is lost due to the coupling efficiency in addition to the 10.5% absorption loss, we estimate $\mu(810\,\mathrm{nm}) \approx 0$. This low scattering loss is consistent with the light primarily occupying the fundamental mode of the fiber. From here we can derive that for $\eta_T = 43\%$, $\eta_C = 48\%$.

For SPDC, we are not able to measure η_S due to the low photon rate, and thus cannot quantify the division of losses between η_C and η_S. However, we can consider that η_C and η_S will likely only differ from that for laser light if the mode content in the fiber is different. If the SPDC does occupy more higher-order modes than laser light, the higher-order modes tend to have high η_S. This would cause a quick depletion of the modes from the fiber leading to minimal contributions to an E2PA signal. Furthermore, higher-order modes have spatial profiles that are less ideal for 2PA. Thus without losing the bulk of the proper physical model, we will operate under the assumption that only the fundamental mode is relevant in the E2PA measurement.

We followed a similar procedure to that used for measuring the transmission efficiency of the laser in order to estimate the number of SPDC spatial modes. Here we write the transmission efficiency as,

$$\begin{aligned}\eta_T &= \frac{Q_{\text{out}}M}{Q_{\text{in}}^{\text{mm}}} = \frac{Q_{\text{out}}}{Q_{\text{in}}} \\ &= \eta_C \eta_A(\lambda = \lambda_e, z = l)\eta_S(\lambda = \lambda_e, z = l).\end{aligned} \tag{4}$$

where M is the total number of spatial modes of the incident light, Q_{in} (photons s^{-1}) and Q_{out} (photons s^{-1}) are the incident and output photon count rate and $Q_{\text{in}}^{\text{mm}}$ (photons s^{-1}) is the incident photon count rates measured for a multimode (mm) light source. Unless specified with the labeling "mm", all other quantities are for a single spatial mode. The values of Q_{out} and $Q_{\text{in}}^{\text{mm}}$ can be measured directly using an sCMOS camera. The incident photon count rate is an estimate for the incident photon count rate of the single mode coupled into fiber. The highest value achieved for the ratio of output photon count rate to incident multimode photon count rate is $Q_{\text{out}}/Q_{\text{in}}^{\text{mm}} \approx 0.058\%$. We can plug in the values of η_S, η_A and η_C determined using a laser source, as well as $Q_{\text{out}}/Q_{\text{in}}^{\text{mm}}$ into Eq. (4) to estimate $M \approx 740$. This estimate for the number of spatial modes serves as a lower bound since up to 16 modes can be coupled into fiber as calculated using Eq. (1) in the main text.

Thus far we have discussed coupling efficiency η_C defined as the ratio of single photons incident on the fiber to single photons output from the fiber—all of which originate from the same single mode. This is in contrast to the Klyshko efficiency[2]—a quantity typically measured for photon pair sources—defined as the probability of detecting a photon conditioned on the successful detection of its partner photon. For this work, we use a theoretical value for the Klyshko efficiency to aid in our estimation of intact photon pair rate in the fiber, Q_{pairs} (photon pairs s^{-1}). We input various parameters of our pump beam, crystal, lenses and fiber into SPDCalc[3] which calculates the overlap integral of three Gaussian spatial modes—one for signal photons, one for idler photons, and one for the collected single mode in fiber—along the length of the crystal and estimates $\eta_K^{'} = 0.94$. This differs from a measured Klyshko efficiency, η_K, because it does not account for any single photon loss between photon pair generation and collection into fiber. We can relate the two by,

$$\begin{aligned}\eta_K &= \eta_K^{'}\eta_F\eta_C \\ &= \frac{2Q_{\text{pairs}}(z=0)}{Q_{\text{singles}}(z=0)},\end{aligned} \tag{5}$$

where η_F is the free-space transmission efficiency between the center of the crystal to the fiber and $Q_{\text{pairs}}(z)$ and $Q_{\text{singles}}(z)$ (photons s^{-1}) are the rates of photon pairs and single photons, respectively, in fiber. In later sections, we will refer to $Q_{\text{singles}}(z)$ simply as $Q(z)$. The factor of 2 is used to align with the standard definition of η_K, which is typically measured using two fibers. In writing this equation and Eq. (4), we have assumed that the efficiencies of the various frequency modes are equal. We find that $\eta_K = 0.25$.

We image the 810 nm laser light at the output of the fiber using two lenses before the sCMOS camera, as shown in Fig. 2(c). In this image, the bright larger and smaller concentric circles are the imaged fiber cladding outer diameter and the outline of the core modes, respectively. The larger offset circular shape is likely formed by light guided through the inside of the tubing sleeve as can be seen by comparison to the digital microscope image in Fig. 2(b). Thus, we

find that the light occupies some lower intensity cladding and tubing sleeve modes in addition to the dominant core modes.

The fluorescence is collected at the front of the fiber using the lens optimized to focus 810 nm light into the fiber, which roughly collimates the visible fluorescence. The light is reflected at the dichroic beamsplitter, and another lens focuses the image of the fiber face onto the EMCCD, as shown in Fig. 2(d). In this image, the high intensity bright spot is from fluorescence guided through the core. Surrounding that spot is darkness likely from the cladding of the fiber, indicating that there is little light propagating through the cladding of the fiber. Radially outward from the cladding, light forms an offset circular shape and is likely from light guided through the core of the tubing sleeve as can be seen by comparison to the digital microscope image in Fig. 2(b). A larger circular ring of light surrounds that core, and is likely from light guided through the "cladding" of the tubing sleeve. Thus, the spatial mode content of the fluorescence in the fiber is more complex than Eq. (1) in the main text assumes, however the highest intensity mode appears to be the fundamental mode of the fiber.

To measure the free-space group delay dispersion (GDD), D_0 (fs^2), accumulated by the laser pulse as it propagates through the optical setup, we use the GDD tuning function of the laser. We increase the dispersion compensation in increments of 500 fs^2 and take C2PA measurements at each step. The step with the maximum C2PA signal corresponds to the value at which the GDD is optimally compensated at the input of the fiber. We measure $D_0 \approx 2000$ fs^2, $D_0 \approx 2000$ fs^2 and $D_0 \approx 4000$ fs^2 for experiments 1, 2 and 3 respectively. For experiments 2 and 3 the free-space dispersion is compensated for using the internal tuning of the laser system. To estimate D_0 accumulated by the SPDC as it propagates through the optical setup, we calculate the GDD of each optical element in its path from the center of the crystal to the entrance of the fiber. We estimate $D_0 \approx 2100$ fs^2. To determine the fiber group velocity dispersion (GVD) β (fs^2 cm^{-1}), a COMSOL simulation is used to model the filled fiber. The low-order modes are solved for. For the fundamental mode, β is identical to β of toluene, thus there are no contributions from waveguide dispersion. However, for higher-order modes it is likely that β is affected by waveguide dispersion. These higher-order modes are not considered in the calculations of the C2PA cross-section and E2PA cross-section upper bound, and β of the fundamental mode is used.

Data Acquisition

In the section we discuss the camera settings used for data acquistion, the characterization of camera baseline and dark count rates, the operations that go into measuring a single frame of data and the conversion of a camera signal to a detected fluorescence rate.

To determine the optimal camera settings, we wrote a script to calculate the expected signal to noise ratio (SNR) of our measurements at various EMCCD settings based on our expected signal levels. We determined the optimum camera settings to be: electron multiplying (EM) output amplifier, EM gain set to 30, preamplifier set to 1, 1 MHz horizontal shift rate, 10 s integration time and 24x24 pixels binned into a superpixel. Although the integration time could be increased further to increase the SNR, we found that clock-induced charge (CIC) occurred more frequently at those integration times. Any obvious CIC was removed from the data, which resulted in a removal of about 6.8% of the frames. To speed up frame readout on the EMCCD, a pixel region of interest (ROI) is selected and only those pixels are read out. To select an ROI, the fluorescence was imaged at a relatively high excitation power and the region with significant photon counts was selected.

Before data series are acquired, the baseline and dark count rates of the camera are characterized. Both are characterized with the built-in camera shutter closed. The baseline data series are taken at the minimum integration time of the camera. The dark rate data series are taken at the 10 s integration time used for all

2PA data series. From each of these characterization data series we calculate an average value and uncertainty using an Allan deviation analysis like that shown in Fig. 4 for the fluorescence rate. An example baseline average in our ROI yields 560.4 ± 1.3 Analog to Digital Units per pixel (ADU pixel^{-1}) and an example dark rate average in our ROI yields 2.66 ± 0.06 electrons s^{-1} pixel^{-1}.

Each two-photon excited fluorescence (2PEF) data frame consists of a background and a signal measurement. The low profile power sensor (Thorlabs S130c) (Fig. 3) is flipped into the beam path to block the beam during a background measurement. For the signal measurement, the power sensor is removed from the beam. We ensured that the power sensor position did not affect the background signal with the laser on. The power sensor after the fiber is used to measure the laser power (W_{out}) before each signal frame and is used to determine W_0 for plotting the power dependence of the signal (Fig. 5(a)). For SPDC data frames, the sCMOS camera is placed at the output of the fiber and used to measure the SPDC power (W_{out}) before each signal frame. The number of data frames acquired at each power varied. As the power decreased, the signal decreased and thus we acquired data for longer to lower the uncertainty.

To calculate the fluorescence rate F in cnt s^{-1} detected by the camera, the pixels of interest are integrated over to determine the camera signal N (ADU). We converted N to a count rate using,

$$F = \frac{NS}{GT}, \tag{6}$$

where S (electrons ADU^{-1}) is the CCD sensitivity for the selected output amplifier and preamplifier, G (electrons cnt^{-1}) is the EM gain and T (s) is the integration time.

Data Normalization

In this section we discuss the data normalization used to account for fluctuations in excitation power. For this, we made use of W_{out}, which is measured for each frame, since it accounts for potential changes in η_C due to laser pointing drifts unlike W_{in}.

For laser excitation, each frame's fluorescence rate $F_{i,\text{raw}}$ (cnt s^{-1}) was adjusted to a normalized fluorescence rate for frame i, $F_{i,\text{norm}}$ (cnt s^{-1}), using

$$F_{i,\text{norm}} = F_{i,\text{raw}} \frac{W^2_{\text{out,avg}}}{W^2_{\text{out,i}}}, \tag{7}$$

where $W_{\text{out,i}}$ (W) is the power transmitted out of the fiber for frame i and $W_{\text{out,avg}}$ (W) is the average power transmitted out of the fiber for the entire data series. Here the normalization uses a quadratic power dependence in accordance with the power scaling of C2PA.

For SPDC excitation, each frame's fluorescence rate $F_{i,\text{raw}}$ was adjusted to a normalized fluorescence rate for frame i using

$$F_{i,\text{norm}} = F_{i,\text{raw}} \frac{W_{\text{out,avg}}}{W_{\text{out,i}}}. \tag{8}$$

Here the normalization uses a linear power dependence in accordance with the expected power scaling of E2PA at sufficiently low power.

For all data series, the normalization has minimal effect and alters the value of the data points by less (typically much less) than one Allan deviation. This indicates that the laser is fairly stable throughout each measurement. The normalization is still used to demonstrate that a high level of characterization and analysis is essential, especially for the SPDC measurement.

For the SPDC measurement, a quadrant detector is used to monitor the pump power while the sCMOS camera is used to measure W_{out}. The output power varied over a range of 24% of the maximum value, and decreased monotonically relative to the pump power by 24% over the course of the 24 hour measurement. The latter value is directly related to a decrease in coupling efficiency. The value $Q_{\text{out}}/Q^{\text{mm}}_{\text{in}} = 0.058\%$ is the average value over the course of the measurement. Similarly, η_C, which is measured using the laser, is the average from three datasets each measured over the course of more than 24 hours and was thus also subject to drifts in coupling efficiency.

Calculating a C2PA Cross-Section

In this section we discuss the assumptions and equations used to calculate a C2PEF signal. We arrive at an equation to derive a C2PA cross-section. Finally, we use these equations to model the concentration dependence of the signal and compare with our results.

In a similar manner to that shown in Ref. 4, we can model the C2PEF signal detected on the camera, F_C (cnt s^{-1}), as

$$F_C = g \int_0^l N_C(z) \int_{\lambda_1}^{\lambda_2} \gamma(z,\lambda)\kappa(\lambda)\Phi(\lambda)d\lambda dz, \tag{9}$$

where g (pulses s^{-1}) is the pulse repetition rate, l (cm) is the length of the fiber, $N_C(z)$ (excitations cm^{-1} pulse^{-1}) is the number of excitations per infinitesimal length of fiber dz (cm) per pulse, λ_1 and λ_2 (nm) are wavelengths chosen such that the integral extends over the entire emission spectrum of the sample, $\gamma(z,\lambda)$ (cnt photon^{-1}) is the component transmission efficiency, $\kappa(\lambda)$ is the geometrical collection efficiency and $\Phi(\lambda)$ (photon excitation^{-1} nm^{-1}) is the differential fluorescence quantum yield. A proper normalization of quantum yield is used such that $\Phi = \int_0^\infty \Phi(\lambda)d\lambda$ gives the value published in literature for the total quantum yield of the fluorophore.

From Eq. (9), we find that it is advantageous to increase the length of the fiber for all z such that $N_C(z)\gamma(z,\lambda) > 0$. This product never drops below zero, but can be zero if the photons are dispersed in time far enough that two photons are never temporally overlapped at that position z, or if the photon loss along the length l is 100% for either the excitation photons or the fluorescence photons, or some combination of both leading to a negligible product $N_C(z)\gamma(z,\lambda) \approx 0$. Since extra fiber length does not decrease the C2PEF signal, we try to make l long enough that $N_C(l)\gamma(l,\lambda) \approx 0$ so that we can achieve the highest signal at a given photon flux.

Here we define $\kappa(\lambda)$ as the fraction of fluorescence that can be collected by the fiber and directed out in the direction of the detector. We assume that the fluorescence is emitted isotropically and that the fraction of that light collected can be described by the solid angle of a cone with apex angle $2(90^\circ - \theta_c)$, where θ_c is the critical angle (see main text). Then we can write,

$$\kappa(\lambda) = \tfrac{1}{2} \times \left[1 - \cos\left(\sin^{-1}\left(\frac{\sqrt{n^2_{\text{core}}(\lambda) - n^2_{\text{clad}}(\lambda)}}{n_{\text{core}}(\lambda)}\right)\right)\right], \tag{10}$$

where $n_{\text{core}}(\lambda)$ and $n_{\text{clad}}(\lambda)$ are the indices of refraction of the core and cladding materials (toluene and silica) at the wavelength of the fluorescence (peak wavelength $\lambda_f = 451$ nm).

The component transmission efficiency describes the transmission efficiency of fluorescence from its point of generation z (cm) to the detector, under the assumption that it is directed out of the fiber. It takes into account all the loss mechanisms from various optical components and the media the light propagates through. We define this quantity as

$$\begin{aligned}\gamma(z,\lambda) &= \eta_A(\lambda,z)\eta_S(\lambda,z)\mathcal{T}_{\text{w}}(\lambda)\mathcal{T}_{\text{l}_1}(\lambda) \times \\ &\quad \mathcal{R}_{\text{d}}(\lambda)\mathcal{T}_{\text{l}_2}(\lambda)\mathcal{T}_{\text{f}_1}(\lambda)\mathcal{T}_{\text{f}_2}(\lambda)\text{QE}(\lambda) \\ &= \eta_A(\lambda,z)\eta_S(\lambda,z)\gamma_0(\lambda),\end{aligned} \tag{11}$$

where $\eta_A(\lambda,z)$ and $\eta_S(\lambda,z)$ are the absorption and scattering efficiencies in fiber (as described earlier), $\mathcal{T}(\lambda)$ and $\mathcal{R}(\lambda)$ are the transmittance and reflectance of an optic (w = window, l = lens, d = dichroic beamsplitter and f = spectral filter), and QE(λ) (cnt photon^{-1}) is the quantum efficiency of the camera.

We describe the number of excitations per infinitesimal length dz per pulse as

$$N_C(z) = \frac{1}{2}\sigma_C n \times \int_{-1/2g}^{1/2g}\int_{-\infty}^{\infty}\int_{-\infty}^{\infty} \phi(x,y,z,t)^2 dx dy dt, \tag{12}$$

where σ_C (1 GM = 10^{-50} cm^4 s photon^{-1} fluorophore^{-1}) is the C2PA cross-section, n (fluorophores cm^{-3}) is the number density of the fluorophores and $\phi(x,y,z,t)$ (photons cm^{-2} s^{-1}) is the photon flux of the laser

beam. The factor of 1/2 carries units of excitations per photons absorbed. The temporal and transverse spatial profiles of the laser beam are approximated by Gaussian distributions. The transverse spatial profile of the light inside the fiber will differ from a Gaussian distribution if the light occupies the higher-order modes of the fiber, but in this calculation we make a few approximations based on the assumption that all the light is in the fundamental mode. The spatial integrals extend from negative infinity to positive infinity and are equivalent to integrals that extend over only the core of the fiber. Assuming the laser is always on, $\phi(x,y,z,t)$ takes the form

$$\phi(x,y,z,t) = \phi_0(z)\mathrm{Exp}\left(-4\mathrm{ln}2\left(\frac{x^2+y^2}{d_0^2}\right)\right) \times \sum_{i=-\infty}^{\infty} \mathrm{Exp}\left(-4\mathrm{ln}2\frac{(t+i/g)^2}{\tau(z)^2}\right), \tag{13}$$

where $\phi_0(z)$ (photons cm^{-2} s^{-1}) is the peak photon flux as a function of z, $\tau(z)$ (fs) is the FWHM pulse duration and d_0 (cm) is the FWHM beam width. We use COMSOL to solve for d_0 by first solving for the effective mode area A_{eff} (cm^2) of the fundamental mode[5] and solving

$$d_0 = \sqrt{\frac{2\mathrm{ln}2 A_{\mathrm{eff}}}{\pi}}. \tag{14}$$

We can define the average photon rate $Q(z)$ (photons s^{-1}) in terms of the photon flux,

$$\begin{aligned} Q(z) &= g\int_{-1/2g}^{1/2g}\int_{-\infty}^{\infty}\int_{-\infty}^{\infty}\phi(x,y,z,t)dxdydt \\ &= \frac{W(z)}{h\nu}, \end{aligned} \tag{15}$$

where $W(z)$ (W) is the average power of the beam as a function of z and $h\nu$ (J) is the average energy of an incident photon. We can also write the power in a form to show its dependence on propagation losses in fiber as described in Eq. (2), which brings about its z-dependence.

We can solve for $\phi_0(z)$ by inputting Eq. (13) into Eq. (15) and integrating over x, y and t,

$$\phi_0(z) = \left(\frac{4\mathrm{ln}(2)}{\pi}\right)^{3/2}\frac{W(z)}{h\nu g d_0^2\tau(z)}. \tag{16}$$

The pulse duration has z dependence because of dispersion and can be described by

$$\tau(z) = \sqrt{\tau_0^4 + (4\mathrm{ln}2)^2(D_0+\beta z)^2}/\tau_0, \tag{17}$$

where D_0 (fs^2) is the GDD accumulated by the pulse before the fiber and β (fs^2 cm^{-1}) is the total GVD accumulated by the light in the fundamental mode of the fiber.

Now we can rewrite Eq. (9) using these equations as

$$F_C = \sqrt{2}\left(\frac{\mathrm{ln}(2)}{\pi}\right)^{3/2}\frac{\sigma_C n W_0^2}{g(h\nu)^2 d_0^2}\times \int_0^l \frac{\eta_A^2(\lambda_e,z)\eta_S^2(\lambda_e,z)}{\tau(z)}\int_{\lambda_1}^{\lambda_2}\gamma(z,\lambda)\kappa(\lambda)\Phi(\lambda)d\lambda dz. \tag{18}$$

where $\lambda_e = 810$ nm.

Table S1: Summary of fixed parameters.

Parameter	unit	Laser	SPDC
g	pulses s^{-1}	8×10^7	
$h\nu$	J	2.45×10^{-19}	
d_0	μm	2.42	
$a_{\mathrm{sol}}(\lambda_e)^a$	cm^{-1}	0.003	
τ_0	fs	110	N/A
β	fs^2 cm^{-1}	1034	
$\epsilon(\lambda_f)^a$	M^{-1} cm^{-1}	4417	
$\kappa(\lambda_f)^a$		0.0146	
Φ	phot excit^{-1}	0.67 [6]	
F^{LB}	cnt s^{-1}	1.00	
η_F		N/A	0.565
$T_{e,0}$	fs	N/A	260
S_0		N/A	2145
$Q(0)$	photons s^{-1}	N/A	1.49×10^8
M		1	740
η_K		N/A	0.25

[a] $\lambda_e = 810$ nm, $\lambda_f = 451$ nm

Table S2: Summary of variable parameters. Experiments 1 and 2 (Exp 1 and 2) use the same fiber (fiber 1) and laser excitation. Experiment 3 (Exp 3), which involves both laser and SPDC excitation, uses another fiber (fiber 2).

Parameter	unit	Exp 1	Exp 2	Exp 3	
				Laser	SPDC
F_C/W_0^2	cnt s^{-1} μW^2	3.14×10^3	3.19×10^4	3.62×10^5	N/A
c	mM	1.95×10^{-2}	1.70×10^{-1}	2.30	
l	cm	37		36	
$\mu(\lambda_e)^a$	cm^{-1}	0.093		0.034	
D_0	fs^2	2000	0	0	2100
$\gamma_0(\lambda_f)^a$		0.669		0.630	
η_T		0.40	0.43	0.47	0.43

^{a} $\lambda_e = 810$ nm, $\lambda_f = 451$ nm

Then we can solve for the C2PA cross-section,

$$\sigma_C = \frac{1}{\sqrt{2}} \left(\frac{\pi}{\ln(2)}\right)^{3/2} \frac{g(h\nu)^2 d_0^2}{n} \frac{F_C}{W_0^2} \times \left(\int_0^l \eta_A^2(\lambda_e, z) \eta_S^2(\lambda_e, z)/\tau(z) \times \int_{\lambda_1}^{\lambda_2} \gamma(z,\lambda)\kappa(\lambda)\Phi(\lambda) d\lambda dz \right)^{-1}. \tag{19}$$

All the parameters in Eq. (19) are known through experiments or simulations. The parameters d_0 and β are estimated using COMSOL Multiphysics Simulation software, F_C/W_0^2 (cnt s^{-1} μW^{-2}) is the fit to our experimental C2PEF data (and W_0 is estimated using measured $W(l)$), n is measured using a spectrophotometer, $a_{\text{sol}}(\lambda)$ is from literature,[1] $\mu(\lambda)$ is determined from scattering measurements, τ_0 is measured using a SwampOptics Grenouille 8-50-USB, D_0 is measured, $k(\lambda)$ is calculated, $\Phi(\lambda)$ is known from published AF455 measurements[6,7] and $\gamma(z, \lambda)$ is calculated based on optics' specifications, our scattering measurements and the published extinction coefficient and spectra of AF455.[7] Table S1 and Table S2 summarize parameter values used in this calculation and that described in the following section.

The uncertainty on derived C2PA cross-sections was determined by propagating the errors of the various parameters that go into Eq. (19). We multiply this value by a coverage factor ($k = 2$) to reach $\approx 95\%$ confidence that the true value lies within the bounds set by the error bars. The uncertainty from each experiment is 34%.

In Figure S2, we plot concentration normalized C2PEF ($\frac{F_C}{c}$) at 100 nW from measurements (fit) and calculations (calc) for the experimental conditions of experiments 1 (blue), 2 (orange) and 3 (green). The calculations use Eq. (18) with the values of parameters input from Tables S1 and S2. The uncertainty on these values is propagated, and multiplied by a coverage factor ($k = 2$) to give a total uncertainty of 38% on the calculation (shown by shaded regions). After each experiment, small improvements to the setup were made that resulted in a shift of the curve upwards on the plot as experiments progressed from 1 to 3. The trend of each curve shows the same decrease of $\frac{F_C}{c}$ as a function of c. This general decrease is due to fluorescence reabsorption by the abundant molecules available at high concentrations. The data points from measurements are extracted using quadratic fits (slope fixed to 2.00) to each experiments' dataset of fluorescence as a function of power. The fit is extrapolated to 100 nW and that value is divided by the measured concentration of the experiment's sample. These data points are within the expected range based on the calculations and their respective uncertainties.

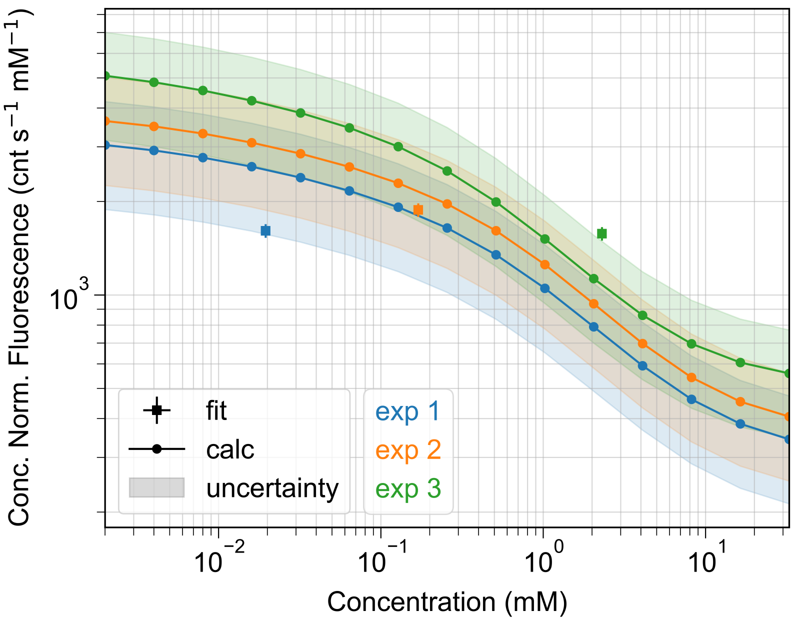

Figure S2: Measured (fit) and calculated (calc) concentration (conc.) normalized (norm.) classical two-photon excited fluorescence at 100 nW excitation as a function of concentration. Experiments 1, 2 and 3 are shown in blue, orange and green respectively. Calculations (using Eq. (18)) were done for each experiment because of minor differences in the setup, which shifts the curve slightly. The calculations predict that as concentration increases there is a general decrease in the concentration normalized fluorescence, which is a result of reabsorption of fluorescence. A shaded region around each of the calculated results show the uncertainty in the calculation. For each experiment, the fit to the measured data was used to extract the concentration normalized fluorescence at 100 nW.

Calculating an E2PA Cross-Section Upper Bound

In this section, in a similar manner to the previous section, we discuss the assumptions and equations used to calculate an E2PEF signal. We arrive at an equation to derive an E2PA cross-section upper bound.

We model the E2PEF signal detected on the camera, F_E (cnt s^{-1}), as

$$F_E = g \int_0^l N_E(z) \int_{\lambda_1}^{\lambda_2} \gamma(z, \lambda)\kappa(\lambda)\Phi(\lambda) d\lambda dz, \tag{20}$$

where $N_E(z)$ (excitations cm^{-1} pulse^{-1}) is the number of excitations per infinitesimal length of fiber dz (cm) per pulse and the other parameters are as mentioned in the previous section. The parameter $N_E(z)$ is defined as

$$N_E(z) = \sigma_E(z) n \frac{Q_{\text{pairs}}(z)}{g} \tag{21}$$

where $\sigma_E(z)$ (cm^2 fluorophore^{-1}) is the E2PA cross-section, which has z dependence due to group delay dispersion broadening the entanglement time and $Q_{\text{pairs}}(z)$ (photon pairs s^{-1}) is the SPDC photon pair rate in fiber. The other parameters are as mentioned in the previous section.

In contrast to C2PA, E2PA requires the presence of spatially and temporally correlated photon pairs. Furthermore the excitation rate scales linearly, as opposed to quadratically, with photon rate in the low-gain regime. In this section, we use the scaling in the low-gain regime to make the most conservative estimate of the upper bound of the E2PA cross-section. In order to write an equation for $Q_{\text{pairs}}(z)$, we will first write the SPDC single photon rate at the fiber, $Q(z)$ (photons s^{-1}),

$$\begin{aligned} Q(z) &= \eta_F \eta_C \eta_A(\lambda_e, z)\eta_S(\lambda_e, z)\frac{Q_{\text{xtal}}^{\text{mm}}}{M} \\ &= \eta_F \eta_C \eta_A(\lambda_e, z)\eta_S(\lambda_e, z) Q_{\text{xtal}}, \end{aligned} \tag{22}$$

where $Q_{\text{xtal}}^{\text{mm}}$ (photons s^{-1}) is the total (all modes) single photon rate at the crystal, M is the number of spatial modes of the generated SPDC, Q_{xtal} (photons s^{-1}) is the single photon rate at the crystal for the spatial mode coupled in fiber, and the other parameters are as mentioned in previous sections.

We can then write the SPDC photon pair rate at the fiber in terms of the single photon rate,

$$\begin{aligned} Q_{\text{pairs}}(z) &= \eta_K \eta_A(\lambda_e, z)\eta_S(\lambda_e, z)\frac{Q(z)}{2} \quad (23) \\ &= \eta_K \eta_F \eta_C \eta_A^2(\lambda_e, z)\eta_S^2(\lambda_e, z)\frac{Q_{\text{xtal}}(z)}{2} \\ &= \eta_K^{'} \eta_F^2 \eta_C^2 \eta_A^2(\lambda_e, z)\eta_S^2(\lambda_e, z)\frac{Q_{\text{xtal}}(z)}{2}, \end{aligned}$$

where $\eta_K^{'}$ is the effective Klyshko efficiency and η_K is the Klyshko efficiency as defined in Eq. (5). The third line of this equation illuminates a signature of E2PA—while E2PA scales linearly with photon rate, it scales quadratically with single photon loss. More details on this signature can be found in Ref. 4.

Here we will use the probabilistic model (see for example Ref. 4) to describe $\sigma_E(z)$ as

$$\sigma_E(z) \approx \frac{\sigma_C}{T_e(z)A_e}, \quad (24)$$

where $T_e(z)$ (fs) is the entanglement time which increases in the presence of group velocity dispersion and thus depends on z and A_e (cm^2) is the entanglement area.

We simulate the entanglement time using a discrete Fourier transform of the measured SPDC joint spectrum shown in Ref. 4. To do this, we estimate the joint spectral amplitude as

$$\begin{aligned} f(\omega_\text{S}, \omega_\text{I}, z) &= \sqrt{F(\omega_\text{S}, \omega_\text{I})} \quad (25) \\ &\times \text{Exp}\left(i(D_0 + \beta z)(\omega_\text{S} - \omega_\text{P}/2)^2/2\right) \\ &\times \text{Exp}\left(i(D_0 + \beta z)(\omega_\text{I} - \omega_\text{P}/2)^2/2\right), \end{aligned}$$

where $F(\omega_\text{S}, \omega_\text{I})$ is the measured joint spectral intensity, and ω_S, ω_I and ω_P are the signal, idler and pump frequencies and the parameters D_0 and β are described in the previous section. Discrete Fourier transforms are performed at discrete steps along the fiber of 1 cm and the entanglement time is calculated as the FWHM of the projection of the joint temporal intensity along the $(t_\text{S} - t_\text{I})$ axis. Here t_S and t_I are the time of arrival of signal and idler respectively. The FWHM is calculated from standard deviation due to the spectrum's non-Gaussian nature. We fit the z-dependence of the resulting T_e values using

$$T_e(z) = 2\sqrt{2\ln 2}\sqrt{T_{e,0}^4 + S_0(\beta z + D_0)^2}/T_{e,0}, \quad (26)$$

where $T_{e,0}$ (fs) and S_0 are fitting parameters and the preceding numerical factors convert standard deviation to FWHM. Physically $T_{e,0}$ should correspond to the entanglement time of the SPDC at the center of the crystal, which is 17 fs, however for our conditions that would render the fit far from ideal. We believe the reason for this is related to the very non-Gaussian form of the spectrum. The parameter S_0 is a numerical factor that is also related to the shape of the spectrum.

In order to estimate a single value for the E2PA cross-section, we will use

$$\sigma_E(0) = \frac{\sigma_C}{T_e(0)A_e}. \quad (27)$$

We can then rewrite Eq. (21) as

$$N_E(z) = \sigma_E(0)n\frac{T_e(0)}{T_e(z)}\frac{Q_{\text{pairs}}(z)}{g}. \quad (28)$$

Then we can rewrite Eq. (20) as

$$\begin{aligned} F_E = \sigma_E(0)n\int_0^l \frac{T_e(0)}{T_e(z)}Q_{\text{pairs}}(z) \\ \times \int_{\lambda_1}^{\lambda_2}\gamma(z,\lambda)\kappa(\lambda)\Phi(\lambda)d\lambda dz. \quad (29) \end{aligned}$$

We can solve for $\sigma_E(0)$,

$$\begin{aligned} \sigma_E(0) = F_E \times \Bigg(n\int_0^l \frac{T_e(0)}{T_e(z)}Q_{\text{pairs}}(z) \\ \times \int_{\lambda_1}^{\lambda_2}\gamma(z,\lambda)\kappa(\lambda)\Phi(\lambda)d\lambda dz\Bigg)^{-1}. \quad (30) \end{aligned}$$

Then for a null measurement, we replace F_E by the measurable fluorescence lower bound F^{LB}

(cnt s^{-1}) and $\sigma_E(0)$ becomes σ_E^{UB},

$$\sigma_E^{\mathrm{UB}} = F^{\mathrm{LB}} \times \left(n \int_0^l \frac{T_e(0)}{T_e(z)} Q_{\mathrm{pairs}}(z) \times \int_{\lambda_1}^{\lambda_2} \gamma(z,\lambda)\kappa(\lambda)\Phi(\lambda) d\lambda dz \right)^{-1}. \quad (31)$$

All the parameters in Eq. (31) are known through experiments or simulations. The parameter F^{LB} is estimated to be 1.0 cnt s^{-1} based on the signal levels that could be distinguished in these measurements, $T_{e,0}$ and S_0 are estimated using the discrete Fourier transform of the measured joint spectrum, D_0 is estimated, $Q_{\mathrm{pairs}}(z)$ is calculated using the measured value of Q_{out} and measured and estimated values of efficiencies, and all other parameters are found as stated in the previous section. The values of these parameters are listed in Tables S1 and S2.

The uncertainty on the derived E2PA cross-section upper bound was determined by propagating the errors of the various parameters that go into Eq. (31) and multiplying this value by a coverage factor ($k = 2$), which gives 40%.

References

(1) Kedenburg, S.; Vieweg, M.; Gissibl, T.; Giessen, H. Linear refractive index and absorption measurements of nonlinear optical liquids in the visible and near-infrared spectral region. *Opt. Mater. Express* **2012**, *2*, 1588–1611.

(2) Klyshko, D. N. Use of two-photon light for absolute calibration of photoelectric detectors. *Sov. J. Quantum Electron.* **1980**, *10*, 1112–1116.

(3) Shalm, L. K. SPDCalc application. `https://app.spdcalc.org/`, (accessed 2025-01-13).

(4) Parzuchowski, K. M.; Mikhaylov, A.; Mazurek, M. D.; Wilson, R. N.; Lum, D. J.; Gerrits, T.; Charles H. Camp, J.; Stevens, M. J.; Jimenez, R. Setting Bounds on Entangled Two-Photon Absorption Cross Sections in Common Fluorophores. *Phys. Rev. Appl.* **2021**, *15*, 044012.

(5) Agrawal, G. P. *Nonlinear Fiber Optics (Sixth Edition)*; Academic Press, 2019; Chapter 2: Pulse propagation in fibers, pp 27–55.

(6) Rogers, J. E.; Slagle, J. E.; McLean, D. G.; Sutherland, R. L.; Sankaran, B.; Kannan, R.; Tan, L.-S.; Fleitz, P. A. Understanding the One-Photon Photophysical Properties of a Two-Photon Absorbing Chromophore. *J. Phys. Chem. A* **2004**, *108*, 5514–5520.

(7) de Reguardati, S.; Pahapill, J.; Mikhaylov, A.; Stepanenko, Y.; Rebane, A. High-accuracy reference standards for two-photon absorption in the 680-1050 nm wavelength range. *Opt. Express* **2016**, *24*, 9053–9066.
